# Dose-, duration- and age-dependent effects of zoledronic acid on bone structure and mechanical properties in growing rice rats

**DOI:** 10.3389/fendo.2026.1772372

**Published:** 2026-06-03

**Authors:** J. Ignacio Aguirre, Jonathan G. Messer, Evelyn J. Castillo, Tatilla L. da Silva Frota Gomes, Carlos A. Urrego, David H. Kohn, Donald B. Kimmel

**Affiliations:** 1 Department of Physiological Sciences, University of Florida (UF), Gainesville, FL, United States; 2 Department of Biomedical Engineering, University of Michigan, Ann Arbor, MI, United States; 3 Department of Biologic and Materials Sciences, University of Michigan, Ann Arbor, MI, United States

**Keywords:** adolescence, antiresorptive, biomechanics, growing skeleton, pQCT, zoledronate

## Abstract

**Background:**

Zoledronic acid (ZOL), a potent bisphosphonate, is increasingly used off-label in pediatric bone disorders, but its long-term effects on the growing skeleton remain underexplored. This study aimed to investigate the impact of ZOL dose, treatment duration, and age at treatment initiation on femoral structure and mechanical properties in growing rice rats, addressing a critical knowledge gap.

**Methods:**

Two complementary preclinical studies were performed in female rice rats. Study 1 examined dose-dependent effects by administering ZOL (0, 8, 20, 50, 125 µg/kg) every 4 weeks, starting at age 4 weeks, for up to 30 weeks. Study 2 investigated age at treatment initiation (4, 16, or 22 weeks) and durations (6, 12, or 18 weeks) using a single oncologic dose of ZOL (80 µg/kg every 4 weeks). Femoral cancellous and cortical pQCT assessed bone mineral content (BMC), volumetric bone mineral density (vBMD), area and microstructure. 3-point bending of the femoral diaphysis was used to determine biomechanical properties.

**Results:**

ZOL increased total metaphyseal BMC, vBMD, and area, with oncologic doses (20-125 µg/kg) producing 15-25% greater increases in metaphyseal BMC than the osteoporosis dose (8 µg/kg) after 18–30 weeks of treatment. Cortical parameters (area and periosteal circumference) and mid-diaphyseal mechanical properties (ultimate load, moment of inertia) also increased when treatment began in young, growing rats and was maintained for 18–24 weeks, although clear dose–response effects were limited. When ZOL started later, skeletal responses were attenuated and required longer treatment durations to become significant.

**Conclusions:**

ZOL enhances metaphyseal and cortical bone properties while improving mechanical strength, particularly when treatment is initiated at an early age. The skeletal response depends on both duration and age at initiation. Notably, ZOL dose-response effects are pronounced in cancellous bone. Cortical structural and mechanical properties exhibit a nonlinear response, with significant differences observed only at extended treatment durations.

## Introduction

Nitrogen-containing bisphosphonates (N-BPs) are a class of drugs that strongly inhibit osteoclast-mediated bone resorption, bone modeling, and bone remodeling (1–3). Their uses and effects are well-established in the adult patient population. Indeed, large-scale randomized controlled trials have validated N-BPs’ effectiveness in enhancing bone mineral density (BMD) and reducing the risk of hip and vertebral fractures by approximately 40-70% in adult patients (4–6).

N-BPs have been used off-label in children and adolescents for decades to treat bone disorders, such as osteogenesis imperfecta, secondary osteoporosis, and other conditions, including cancer, to reduce fragility fractures, prevent excessive bone loss, improve BMD, prevent bone metastases, and relieve pain (7–15). Their off-label use in pediatrics remains controversial due to limited long-term efficacy and safety data (7, 16, 17). Because N-BPs suppress bone modeling and remodeling, processes essential for normal skeletal growth and maturation, their use in growing patients carries potential risks. Suppression of bone modeling and remodeling may negatively impact bone growth, compromise skeletal integrity, and have long-term effects on bone quality, bone morphology, overall skeletal health, and development (17).

Zoledronic acid (ZOL), a third-generation N-BP, is commonly used in adult patients but has not been formally approved for pediatric use in the United States and Europe (12, 18, 19). Extensive research has focused on the effects of N-BPs on bone architecture, biomechanical properties, and the quality of the mature skeleton in adults, especially the elderly (20–26). However, comparable data for the growing skeleton is limited. Despite this, physicians prescribe ZOL when they believe the potential benefits outweigh the risks for certain pediatric conditions. In this respect, studies have demonstrated the effectiveness during childhood of ZOL for secondary osteoporosis, osteogenesis imperfecta (OI), metastatic bone disease, hypercalcemia, avascular necrosis, chronic recurrent multifocal osteomyelitis, fibrous dysplasia, McCune-Albright Syndrome, Fibrodysplasia *Ossificans Progressiva*, heterotopic calcification, and chemotherapy-related osteonecrosis (11, 12, 27–31). ZOL dosing in children is largely derived from uncontrolled or case series studies in OI and pediatric osteoporosis. It has been refined to balance efficacy with safety (e.g., starting at 12.5 µg/kg IV to mitigate adverse effects, such as hypocalcemia) (32–37). Typical osteoporosis protocols in pediatrics use age- and response-based escalation from 12.5 to 50 µg/kg IV, given at 3- to 6-monthly intervals (often totaling ~100 µg/kg/year), with dose adjustments guided by lumbar spine BMD Z scores. A common practice is an annual total dose of 0.1 mg/kg, administered as two IV doses of 0.05 mg/kg. ZOL is also used off-label in children for hypercalcemia, with case reports reporting dosages of 25 µg/kg, 50 µg/kg, or a single 4 mg dose (for older children), and one instance of a cumulative 100 µg/kg given over 3 days (12). For malignant bone tumors and metastatic bone disease, retrospective studies show that “multiple high doses” of 4 mg in patients over 10 years old have been well tolerated, improving pain and stabilizing bone disease (12). However, optimal dosing remains under investigation, and individualized clinical judgment is required, particularly outside the context of osteogenesis imperfecta.

It is logical to expect that higher doses of N-BPs given over extended periods would exert a more pronounced effect on bone structure and biomechanical integrity than lower doses used to treat osteoporosis. However, the dose-response relationship of N-BPs like ZOL on bone structure and strength, as well as the differential effects of treatment duration and age at initiation of treatment, have not been directly investigated in the growing skeleton. We hypothesized that ZOL differentially modulates femoral structure and biomechanical strength in growing rice rats (Oryzomys palustris), with effects contingent on the interplay among cumulative dose, treatment duration, and age at initiation. We conducted two complementary preclinical studies in rice rats to evaluate how osteoporosis- and oncology-relevant dose ranges of ZOL, treatment duration, and age at treatment initiation affect the cortical and cancellous bone compartments of the femur.

## Materials and methods

### Justification for the use of the rice rat (*Oryzomys palustris*)

The rice rat is a valuable rodent species for biomedical research. It was initially established as a model for studying specific oral-dental conditions, particularly periodontitis and medication-related osteonecrosis of the jaw (MRONJ) (38, 39). A well-established colony at the University of Florida has been maintained for over 15 years (40). Previous research has not only characterized the species’ fundamental skeletal properties (41) but also demonstrated responsiveness to antiresorptive and bone anabolic agents comparable to that seen in humans and other rodent models (39, 41), underscoring its relevance for bone research.

The bone specimens used in the studies presented here were obtained from comprehensive, larger investigations of the pathophysiology of MRONJ (42), thereby leveraging existing resources and supporting the reduction principle of the 3Rs.

### Animal care

A monogamous, continuous-breeding system was used to generate rice rat pups for the studies (40). At weaning (age 4 weeks), clinically normal female rice rats [body weight (BW) ≥ 30g and body condition score (BCS) ≥ 3.0] were randomized into the experiments, with efforts made to distribute littermates across different groups (42). Breeder rats ate rodent breeder chow (Envigo Teklad, irradiated 2919). Experimental animals ate a standard (STD) diet [Envigo Teklad LM-485 (irradiated 7912) Rodent Diet; Tampa, FL, USA]. BW was measured bi-weekly. All rats were housed (2–5 rats per cage) in static filter top cages (area: 143 in^2^) with pine shavings as bedding and continuous access to food and water. The housing room was maintained at 68-79°F, with an average humidity of 30-70%, and a 12:12hr light: dark cycle. Breeder pairs were housed under the same conditions as the experimental rats, but with a 14:10-hour light: dark cycle. The Animal Care Services program at the University of Florida (UF) is AAALAC-accredited. Both protocols were approved by the UF Institutional Animal Care and Use Committee (IACUC).

### Study design

Two complementary studies were undertaken to test the proposed hypothesis. For consistency and enhanced translational context, the developmental stages of the female rice rats used here were systematically categorized based on specific week ranges, each assigned an approximate human age equivalent. These stages are defined as: juvenile (4–8 weeks), broadly corresponding to human adolescence; young adulthood (8–18 weeks), broadly corresponding to human young adulthood; middle adulthood (18–28 weeks), comparable to human mid-adulthood; and mature adulthood (28–40 weeks), generally reflecting human mature adulthood. These definitions were applied throughout both Study 1 and Study 2.

In Study 1 (**Supplementary Figure 1A**), we examined the long-term effects of ZOL dose and treatment duration on the femoral structure, bone quantity, and biomechanical properties of growing rice rats from weanling (age 4 weeks) to adulthood. BW randomly assigned a total of 227 four-week-old female rice rats into a baseline group (n=11; euthanized at 4 weeks of age) and five ZOL treatment groups (n=42-44/group). Rice rats received intravenous (IV) injections every four weeks (q4wks) with either saline (VEH; 0 µg/kg ZOL) or ZOL at 8, 20, 50, or 125 µg/kg. The 8 µg/kg dose corresponds to the established osteoporosis dose in rice rats (42, 43). The ZOL doses were increased step-wise by a factor of 2.5-fold, with the 20–125 µg/kg ZOL doses representing oncology-relevant exposures, and rats were randomly assigned for euthanasia at 12, 18, 24, or 30 weeks (n=9–12 per subgroup) to evaluate dose–response effects over time (42, 43). For malignant bone tumors, including metastatic bone disease, retrospective studies indicate that “multiple high doses” of 4 mg in patients >10 years of age have been well-tolerated and can improve pain control and stabilize bone disease. However, there remains a significant paucity of data regarding its skeletal effects in children (12).

Therefore, in Study 2 (**Supplementary Figure 1B**), we examined how ZOL’s skeletal effects vary as a function of age at treatment initiation and treatment duration, using a clinically relevant oncologic dose of 80 μg/kg (42, 43). To address this, 172 female rice rats were randomized by BW at 4 weeks of age into seven groups. One group (n=12) served as a baseline control and was euthanized at 4 weeks of age. The remaining six groups received IV injections of either ZOL or vehicle (saline) q4 wks. Treatment was initiated at age 4, 16, or 22 weeks, with rat subgroups euthanized after 6, 12, or 18 weeks of ZOL exposure to assess the influence of initiation age and treatment duration. These groups are described as follows: Group 1 rice rats (VEH; n = 32) received IV saline starting at 4 weeks of age and were euthanized after 6, 12, or 18 weeks of treatment (n = 10–12 per subgroup); this group served as the VEH control for Group 2. Group 2 rice rats (ZOL early treatment initiation; n = 31) received ZOL at 80 μg/kg IV q4 wks beginning at 4 weeks of age and were euthanized after 6, 12, or 18 weeks of treatment (n = 10–11 per subgroup). At 16 weeks of age, Group 4 rice rats (ZOL intermediate treatment initiation; n = 39) began ZOL at 80 μg/kg IV q4 wks and were euthanized after 6, 12, or 18 weeks of treatment (n = 12–15 per subgroup). Group 3 (VEH; n = 29) served as the VEH control for Group 4. In accordance with the 3Rs principle of reduction, the 22-week VEH subgroup was shared between Group 1 and Group 3 to minimize the total number of animals used. The remaining VEH subgroup rats in Group 3 received IV saline q4 wks and were euthanized at 28 and 34 weeks of age (n = 7–12 per time point). At 22 weeks of age, Group 6 (late treatment initiation; n = 31) began the oncologic ZOL regimen (80 μg/kg IV q4 wks) and was euthanized after 6, 12, or 18 weeks of treatment (n = 7–12 per subgroup). Group 5 (VEH; n = 25) served as the VEH control for Group 6 (n = 7–8 per time point). To further reduce animal use, the 6- and 12-week VEH subgroups in Group 5 served as the shared controls for the rats euthanized at 28 and 34 weeks of age in Group 3, respectively. Recognizing that age is a critical variable influencing bone parameters, we independently characterized age-related variations in pQCT and biomechanical parameters within the VEH control groups (**Supplementary Figures 2**–**4**). The primary purpose was to establish a baseline of normal growth and age-related changes, enabling us to distinguish age-related effects from ZOL-related effects. Because rats are influenced both by ZOL and age, and because variation in age at treatment initiation and duration changes affects rat ages at necropsy, these baselines are essential for attributing observed differences to ZOL rather than to aging.

### Rationale for the doses of ZOL

In this study, we employed a dose-gradient strategy for ZOL. Intravenous injections of ZOL, dissolved in normal saline (pH 7.2) at 1 ml/kg body weight, were administered to achieve a dose range of 8 to 125 µg/kg. The osteoporosis dose was defined as 8 µg/kg IV monthly, a concentration previously shown to prevent bone loss in ovariectomized models (44–46). Based on the established 10-fold human oncologic-to-osteoporosis dose ratio, the calculated oncologic dose for rats is 80 µg/kg monthly (42, 43, 45, 47). To comprehensively evaluate ZOL’s effects across a broad clinically relevant range, the experimental dose series for Study 1 included 8 µg/kg, 20 µg/kg, 50 µg/kg, and 125 µg/kg. The rationale for these doses and their approximate 2.5-fold incremental increases was a strategic, pragmatic choice, systematically applied to establish a wide dose gradient based on our previous studies (42, 43, 47). This approach allowed us to span a wide spectrum of ZOL exposures, progressing from the osteoporosis-level dose (8 µg/kg) to multiple oncologic doses (20, 50, 125 µg/kg). The highest dose of 125 µg/kg represents a concentration historically used in early human trials for hypercalcemia of malignancy and bone metastasis, which, although discontinued in current clinical practice, provided insights into responses at elevated exposure levels (42, 43, 47). For Study 2, a single intermediate oncologic dose of 80 µg/kg body weight was used.

### Euthanasia and collection of femurs

Rats were euthanized by CO_2_ inhalation followed by cervical dislocation. Femurs were first disarticulated from the acetabulum and then separated intact from the tibia. All muscles and connective tissues were then removed from the femur without damaging the bone itself. Finally, the femur was wrapped in saline-soaked gauze and stored at -20 °C in propylene tubes until subsequent pQCT analysis and bone biomechanical testing (see below).

### Peripheral quantitative computed tomography analysis

pQCT analysis was performed on femurs as previously described, with the operator blinded to animal identity during scanning and analysis (48, 49). Briefly, two days prior to pQCT assessment, samples were transferred to a refrigerator at 4 °C. On the day of pQCT and subsequent mechanical testing, each femur, within its protective tube, was placed in a 37 °C water bath for at least 30 minutes to equilibrate to physiological temperature. For pQCT scans, bones were removed from their saline-soaked gauze for imaging and immediately returned to their hydrated state in the gauze within their tubes after each scan. pQCT scans of the femurs were conducted using a Stratec XCT Research M instrument (Norland Medical Systems; Fort Atkinson, WI), using the company’s software version 5.40. General scan parameters included a voxel size of 0.1 mm, a scan velocity (SV speed) of 30 mm/s, and a CT speed of 4 mm/s. Scans were conducted at two sites: the distal metaphysis (25% of the average femur length) to target the secondary spongiosa, and the mid-diaphysis (50% of the average femur length) to assess cortical bone. BMC, vBMD, and bone area were determined for total bone (trabecular and cortical bone) at the distal metaphysis and mid-diaphysis as previously described (49). For both scan sites, initial segmentation employed global density thresholds of 169 mg/cm³ (to separate soft tissue from bone) and 655 mg/cm³ (to distinguish cortical from trabecular bone components). At the distal femoral metaphysis (trabecular-rich site), the analysis employed contour mode 2 and peel mode 1. Trabecular bone area was defined as 30%. Cortical bone analysis within this region utilized cortical mode 4 with a cortical density threshold of 529 mg/cm³. At the femoral mid-diaphysis (cortical-rich site), the analysis employed contour mode 2 and peel mode 2. Cortical bone parameters were determined using cortical mode 4 with a primary cortical density threshold of 340 mg/cm³ and a secondary threshold of 529 mg/cm³. To simplify the presentation of similar trends, metaphyseal bone data was reported as total rather than being separated into trabecular and cortical compartments.

**Figure 1:**
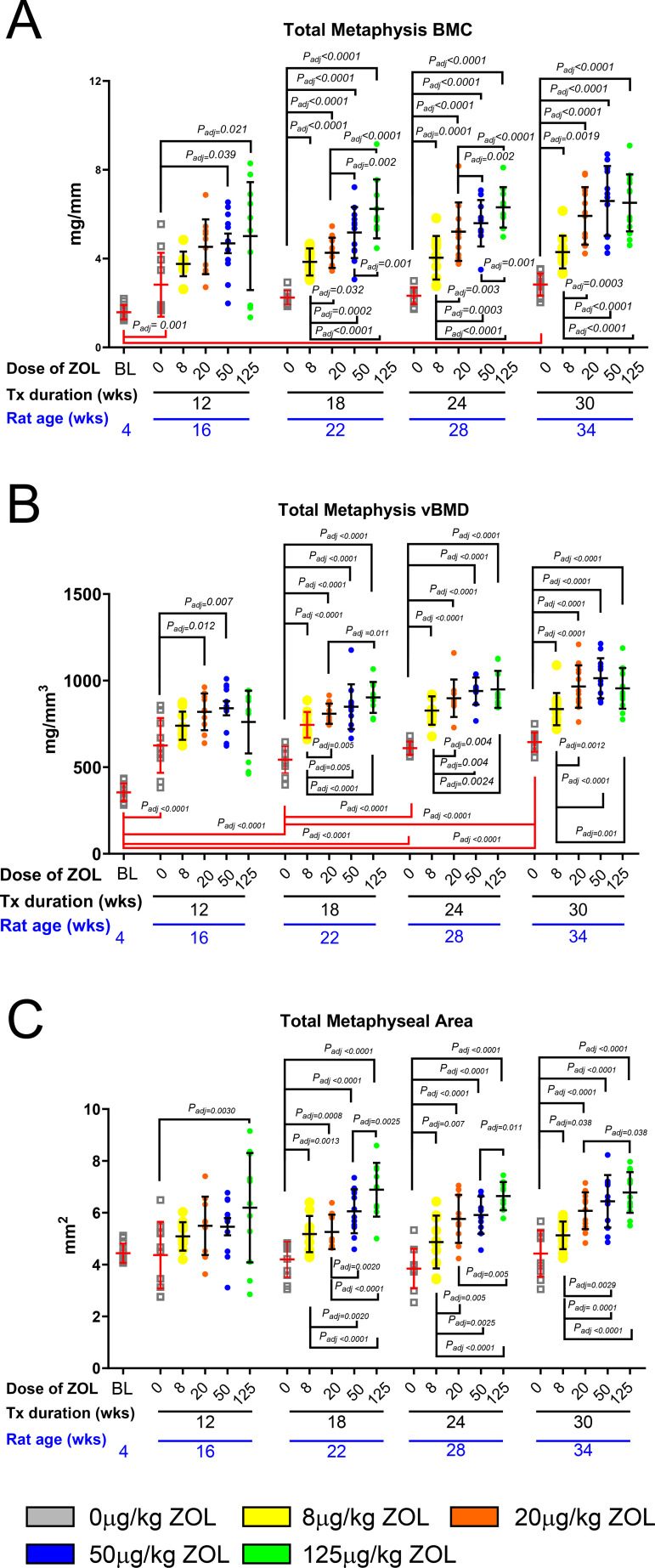
pQCT measurements at the distal femoral metaphysis in rice rats treated with various doses and durations of zoledronic acid (ZOL). Rats (n = 10–15/group) received IV (tail vein) injections q4 weeks of ZOL at 0, 8, 20, 50, or 125 µg/kg starting at age 4 weeks and were necropsied after 12, 18, 24, or 30 weeks. **(A)** total bone mineral content (BMC), **(B)** total volumetric BMD (vBMD), and **(C)** total metaphyseal area. Individual rats are plotted as colored points by ZOL dose. Group means ± SD are shown. Treatment-duration subgroups are indicated in black. Age subgroups are indicated in blue beneath each panel; baseline (BL): age 4 weeks. One-way ANOVA with post hoc Benjamini, Krieger, and Yekutieli (BKY) comparisons procedure. P ≤ 0.05 is considered statistically significant. Brackets denote pairwise comparisons: red: VEH groups of different ages; black: ZOL dose group comparisons at each time point.

**Figure 2:**
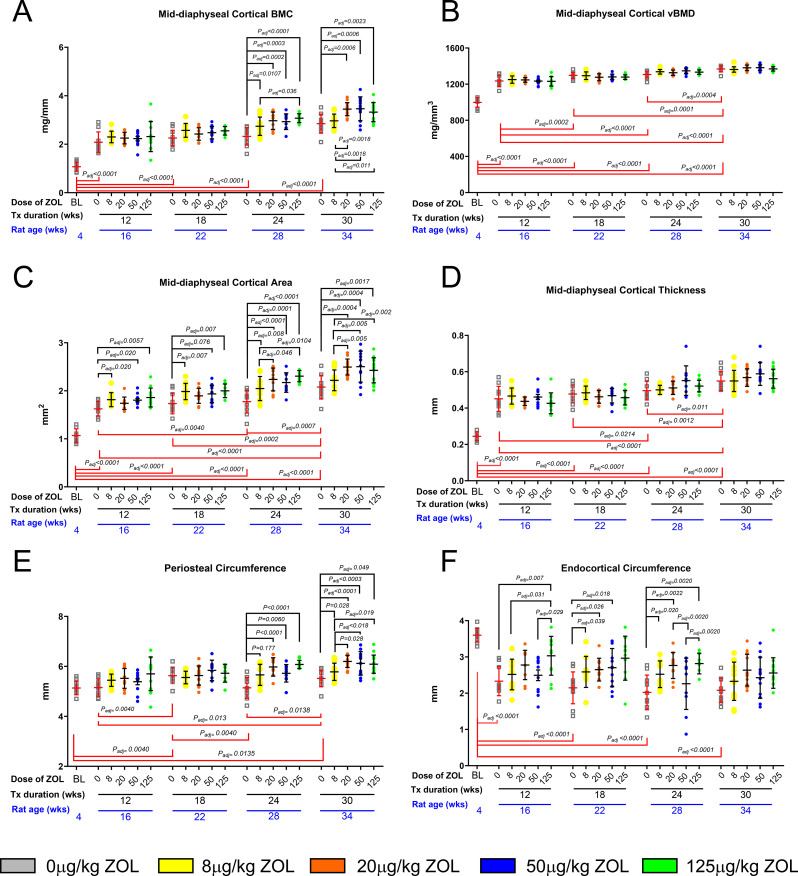
pQCT measurements at the femoral mid-diaphysis in rice rats treated with various doses and durations of zoledronic acid (ZOL). Rats (n = 10–15/group) received ZOL 0, 8, 20, 50, or 125 µg/kg IV (tail vein) every 4 weeks starting at age 4 weeks and were necropsied after 12, 18, 24, or 30 weeks. **(A)** mid-diaphyseal cortical bone mineral content (BMC), **(B)** cortical volumetric BMD (vBMD), **(C)** mid-diaphyseal cortical area, **(D)** mid-diaphyseal cortical thickness, **(E)** periosteal circumference, and **(F)** endocortical circumference. Individual rats are plotted as colored points by ZOL dose, with bars representing group means ± SD. Treatment-duration subgroups are indicated in black, and age subgroups are indicated in blue beneath each panel; baseline (BL): age 4 weeks. One-way ANOVA with *post hoc Benjamini, Krieger, and Yekutieli* (BKY) comparisons procedure. P ≤ 0.05 is considered statistically significant. Brackets denote pairwise comparisons: red, VEH groups of different ages; black, ZOL dose group comparisons at each time point.

The total distal metaphyseal and mid-diaphyseal cortical BMC and vBMD (**Figures 1A, B**, **2A, B**) data were previously shown in **Supplementary Figure 3** of a prior manuscript to verify ZOL treatment efficacy (42). Portions of **Figures 1** and **2**, specifically **Figures 1A, B** and **2A, B**, which correspond to the distal femoral metaphysis (trabecular BMC and vBMD) and mid-diaphyseal cortical (BMC and vBMD) data, reproduce pQCT results previously presented in a Supplementary figure of a prior study (42) solely to verify ZOL treatment efficacy. All other panels in **Figures 1** and **2** are novels, and copyright permission to republish these data was obtained from the original publisher.

### Bone biomechanical properties

Following pQCT scanning, femurs were kept hydrated and tested in three-point bending at room temperature. Each bone was positioned horizontally, with the anterior surface upward, centered on the lower supports and spaced by 9.6 mm. Load was applied perpendicular to the midshaft at the longitudinal midpoint using a 225 N load cell. Tests were conducted to failure at a constant displacement rate of 0.05 mm/s, with data sampled at 2500 Hz to produce load–displacement curves. Load–displacement data were analyzed (WinTest^®^) to obtain stiffness (N/mm), ultimate load (N), work to fracture (mJ; reported as non–area−normalized energy to failure), moment of inertia (mm4), bending moment (N·mm), ultimate stress (MPa), and elastic modulus (MPa). Geometric properties (outer radius, inner radius, and cross−sectional moment of inertia) were calculated from pQCT-derived periosteal and endocortical circumferences assuming a circular midshaft cross section; the derived radii were then used with standard three-point bending equations to compute ultimate stress and elastic modulus. Bivariate Spearman correlations (r) were calculated between pQCT variables (cortical area, cortical thickness, mid−diaphyseal vBMD) and biomechanical outcomes (stiffness, ultimate load). The interpretation of Spearman’s correlation coefficient, R, was based on previous studies (50).

### Statistical analysis

Data is expressed as mean ± SD for each group. The specific analysis plans for Study 1 and Study 2 are shown in **Supplementary Table 1** and described as follows. In Study 1, group comparisons were conducted using a one-way analysis of variance (ANOVA). When global ANOVA was significant, *post hoc* multiple comparisons were performed to identify specific group differences. Five one-way ANOVAs were conducted for each variable. The first compared the baseline group with the VEH groups (0 µg/kg ZOL) from rats aged 16, 22, 28, and 34 weeks. The second group of rats received 0, 8, 20, 50, or 125 µg/kg ZOL q4wks and were necropsied at 16 weeks. The third, fourth, and fifth ANOVAs compared these same dose groups necropsied at 22, 28, and 34 weeks, respectively. To control the false discovery rate (FDR) arising from ~80 comparisons, we used the two-stage linear step-up procedure of Benjamini, Krieger, and Yekutieli (BKY), which is known to have higher power when many tests are performed. The FDR was controlled at P ≤ 0.05, and adjusted P-values (*P adj ≤ 0.05*) were considered statistically significant.

To assess the strength and direction of linear relationships between pQCT and biomechanical strength variables, we used Spearman rank correlation testing due to the non-linear spacing between ZOL doses in GraphPad Prism 10.4.2, with two-tailed tests to determine P values. Interpretation of Spearman’s correlation coefficient (R) followed a previously published classification (50): 0.90–1.00 (very high), 0.70–0.90 (high), 0.50–0.70 (moderate), 0.30–0.50 (low), and 0.00–0.30 (negligible). In addition, a few single linear regression analyses were conducted. All statistical analyses were conducted, and graphs were prepared using GraphPad Prism 10 (GraphPad Software Inc., USA).

In Study 2, data analysis involved separate procedures for VEH groups and for comparisons of ZOL-treated groups with their corresponding VEH controls. For VEH control group comparisons across different time points and ages at treatment initiation, one-way ANOVA was used, followed, as needed, by the Holm–Šidák *post hoc* test for specific intergroup comparisons. ANOVA assumptions of normality were assessed; when these were not met, the nonparametric Kruskal–Wallis test was applied, followed by Dunn’s *post hoc* test for intergroup comparisons. **Supplementary Figures 2**–**4** show the graphs comparing VEH rats necropsied at 4, 10, 16, 22, 28, 34, and 40 weeks of age for each variable. For comparisons involving the ZOL groups, the effects of age at treatment initiation and treatment duration were evaluated by direct pairwise comparisons between each ZOL group and its matched VEH control group.. For each age at initiation (4, 16, or 22 weeks), three t tests were performed per variable, comparing VEH with ZOL after 6, 12, and 18 weeks of treatment, yielding nine t tests per variable. Because the assumption of equal variances could not be met, Welch’s t-test, a more robust test than Student’s t-test under variance heterogeneity, was used. To further limit the overall Type I error rate across all tests, a conservative significance threshold was prespecified: P ≤ 0.01 was considered statistically significant. The rationale for using Welch’s t-test instead of a two- or three-way ANOVA was that skeletal outcomes depend strongly on rat age, making age at treatment initiation and treatment duration non-independent factors that directly affect necropsy age and, in turn, bone developmental stage. This interdependency would confound a multi-way ANOVA, as ZOL group differences in pQCT and biomechanical parameters could reflect age-related growth rather than ZOL treatment effects. We also note that several VEH control groups were used twice during the analyses in order to reduce animal numbers and apply the principle of the three R’s.

## Results

All animals completed each study uneventfully. No significant clinical issues associated with signs of pain/distress were observed. From weaning (4 weeks of age) through adulthood, female rice rats showed normal growth, characterized by a sustained increase in BW and long-bone length. BW data for all groups in Studies 1 and 2 are provided in **Supplementary Tables 2** and **3**, respectively. The rat developmental stages (adolescence, young adulthood, middle adulthood and mature adulthood) have been defined in Materials and Methods.

### Study 1

As with other rodents, pQCT and biomechanical variables in rice rats are affected by age (48). Therefore, we characterized age-related variability in pQCT and biomechanical parameters in VEH (0 µg/kg)-treated female rice rats aged 4 to 34–40 weeks (42).

#### Femoral pQCT and biomechanical variables in VEH-treated rice rats

##### pQCT variables at the distal femoral metaphysis of VEH-treated female rice rats

Total metaphyseal BMC increased from age 4 to 16 weeks and remained stable until age 34 weeks (**Figure 1A**). Total metaphysis vBMD increased from age 4 to 16 weeks, then from age 22 to 28 weeks, and remained stable thereafter (**Figure 1B**). No significant differences in total metaphyseal area were observed during ages 4–34 weeks (**Figure 1C**).

##### pQCT variables at the femoral mid-diaphysis of VEH-treated female rice rats

Cortical BMC increased from age 4 to 16 weeks and remained stable until age 34 weeks (**Figure 2A**). Furthermore, there were significant increases in cortical vBMD, area and thickness from age 4–34 weeks, particularly from age 4 weeks to age 16 weeks, with the highest values observed in rats aged 34 weeks (**Figures 2B–D**). Thus, at age 34 weeks, rats had greater cortical vBMD, area and thickness than at ages 4, 16, 22, and 28 weeks (**Figures 2B–D**). Periosteal circumference increased from age 4 to 34 weeks (**Figure 2E**). In contrast, endocortical circumference decreased from 4 to 16 weeks of age, then remained at that level through 34 weeks of age (**Figure 2F**).

##### Biomechanical variables at the femoral mid-diaphysis in VEH-treated rice rats

###### Structure-level properties

**Figure 3:**
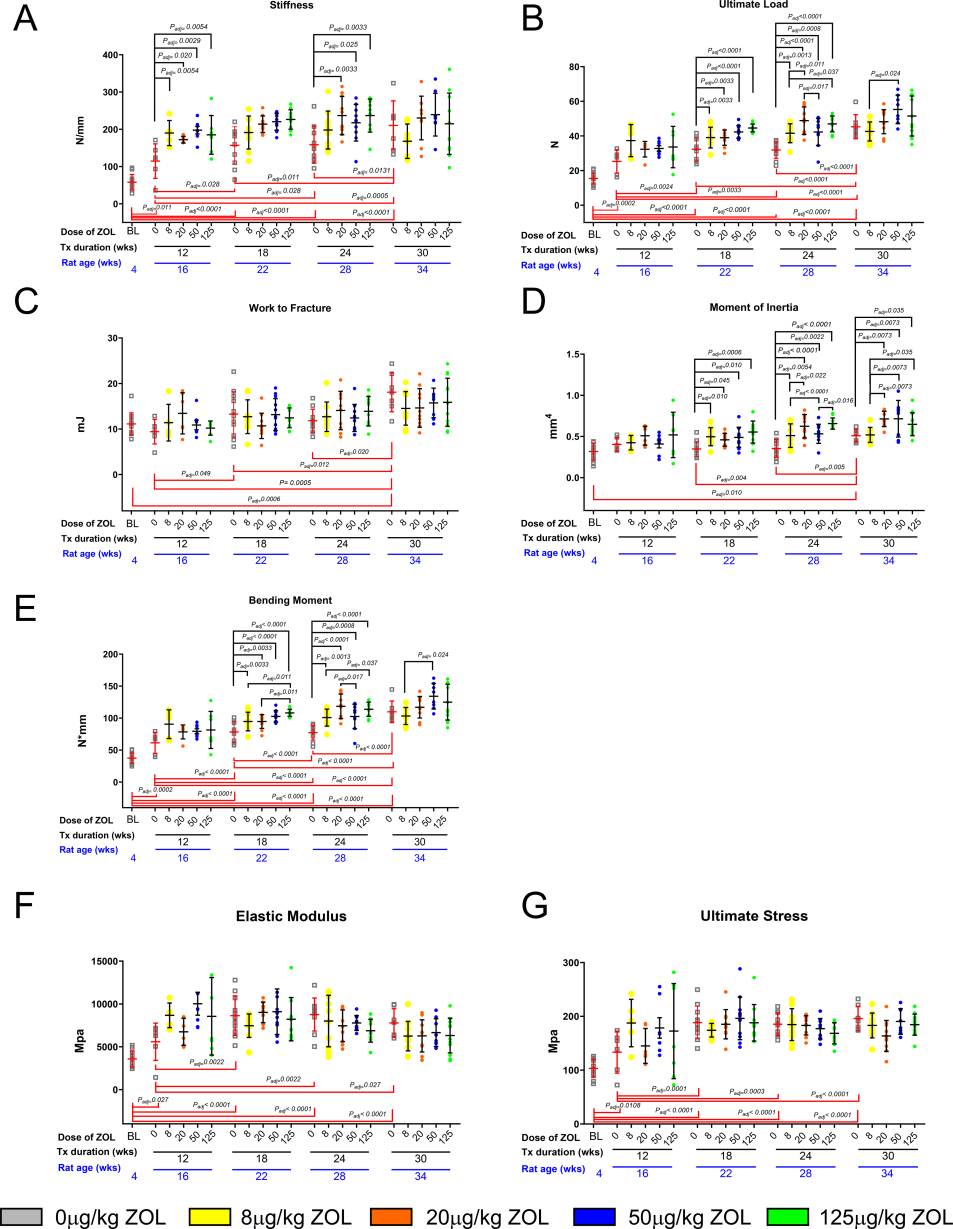
Biomechanical properties at the femoral mid-diaphysis in rice rats treated with various doses and durations of zoledronic acid (ZOL). Rats (n = 10–15/group) received ZOL 0, 8, 20, 50, or 125 µg/kg IV (tail vein) every 4 weeks starting at age 4 weeks and were treated for 12, 18, 24, or 30 weeks. Panels show: **(A)** stiffness (N/mm), **(B)** ultimate load (N), **(C)** work to fracture (mJ), **(D)** moment of inertia (mm^4^), **(E)** bending moment (N*mm), **(F)** elastic modulus (MPa), and **(G)** ultimate stress (MPa). Individual animals are plotted as colored points by ZOL dose. Group means ± SD are shown. Treatment-duration subgroups are indicated in black, and age subgroups are indicated in blue beneath each panel; baseline (BL): 4 weeks. One-way ANOVA with post hoc Benjamini, Krieger, and Yekutieli (BKY) comparisons procedure. P ≤ 0.05 is considered statistically significant. Brackets denote pairwise comparisons: red, VEH groups of different ages; black, ZOL dose group comparisons at each time point.

Stiffness, ultimate load and bending moment steadily increased from age 4 to 34 weeks (**Figures 3A, B, E**). Furthermore, at age 34 weeks, rats had greater work-to-fracture (**Figure 3C**) and greater moment of inertia than at ages 4, 16, and/or 22 weeks (**Figure 3D**).

###### Tissue-level properties

Elastic modulus and ultimate stress increased from age 4 to 16 weeks, then from age 16 to 22 weeks and remained stable until age 34 weeks (**Figures 3F, G**).

#### Femur pQCT and biomechanical variables in rats treated with different doses and durations of ZOL

##### pQCT variables at the distal femoral metaphysis of ZOL-treated rice rats

Overall, increases in total metaphyseal BMC, vBMD, and area became clearly apparent after 18 weeks of ZOL treatment (**Figures 1A–C**). When ZOL was administered for 12 weeks, only a few selected doses (20, 50 and/or 125µg/kg ZOL) resulted in greater metaphyseal BMC, vBMD, or area than VEH rats (**Figures 1A–C**). In contrast, when ZOL treatment durations were extended for 18 weeks or beyond (18–30 weeks), every dose tested (8–125 µg/kg) significantly increased these variables compared with VEH rats (0 µg/kg) (**Figures 1A–C**). Greater increases were also observed in metaphyseal BMC, vBMD, and area when rats were treated with oncologic doses (20–125 µg/kg) compared to the osteoporosis dose (8µg/kg), though the dose response tended to plateau at the 20 µg/kg dose (**Figures 1A–C**).

##### pQCT variables at the femoral mid-diaphysis of ZOL-treated rats

After 12 and 18 weeks of treatment, ZOL effects were evident only for cortical area, with increases in the 8, 50, and 125 µg/kg ZOL groups compared with VEH (0 µg/kg), and for endocortical circumference, for which selected oncologic doses had greater values than VEH or the 8µg/kg rat groups (**Figures 2C, F**).

After 24 weeks of ZOL treatment, cortical BMC, area and periosteal and endocortical circumferences were significantly higher, with nearly all ZOL dose groups showing higher values than VEH rats (**Figures 2A, C, E, F**). Furthermore, a few selected oncologic doses induced greater cortical BMC, area and endocortical circumference than the osteoporosis dose (**Figures 2A, C, F**). After 30 weeks, rats treated with oncologic doses of ZOL (20, 50, 125 µg/kg) had greater BMC and cortical area than rats receiving VEH and the osteoporosis dose (8 µg/kg) (**Figures 2A, C, E**). Rats treated with any ZOL dose (8, 20, 50, 125 µg/kg) had greater periosteal circumference than VEH rats (**Figure 2E**). Furthermore, ZOL treatment did not affect endocortical circumference after 30 weeks of treatment (**Figure 2F**). In addition, none of the ZOL doses or treatment durations affected mid-diaphyseal vBMD or cortical thickness (**Figures 2B, D**).

##### Biomechanical variables at the femoral diaphysis of ZOL-treated rats

###### Structure-level properties

After 12 weeks of treatment, ZOL increased stiffness without affecting any other properties (**Figure 3**). Specifically, stiffness was higher in all ZOL dose groups than in VEH rats (**Figure 3A**). No differences in stiffness between ZOL-treated and VEH rats were observed after 18 weeks of treatment. Then, after 24, but not 30, weeks, only the oncologic doses showed increased stiffness.

All ZOL-treated rats had greater ultimate load, moment of inertia and bending moment than VEH rats at 18 and 24 weeks of treatment (**Figures 3B, D, E**). Rats treated with oncologic ZOL doses also had greater moment of inertia than VEH rats after 30 weeks of treatment (**Figure 3D**).

In addition, several of the higher oncologic ZOL dose rat groups showed higher ultimate load, moment of inertia, and bending moment than the lower oncologic doses or the osteoporosis dose groups after specific 18-, 24-, and/or 30-week treatment intervals (**Figures 3B, D, E**). None of the ZOL doses significantly affected work-to-fracture at any treatment duration (**Figure 3C**).

###### Tissue-level properties

Similarly to work-to-fracture, none of the ZOL doses or treatment duration regimens significantly affected elastic modulus or ultimate stress (**Figures 3F, G**).

###### Spearman’s correlation analysis of ZOL dose levels (0-125µg/kg) with selected pQCT and biomechanical properties

In the analysis, we treated ZOL dose as a quantitative variable to assess the overall dose–response relationship between exposure and bone properties. The VEH group was included to provide a true baseline and to capture changes across the full exposure range, from untreated controls to the highest dose.

**Figure 4:**
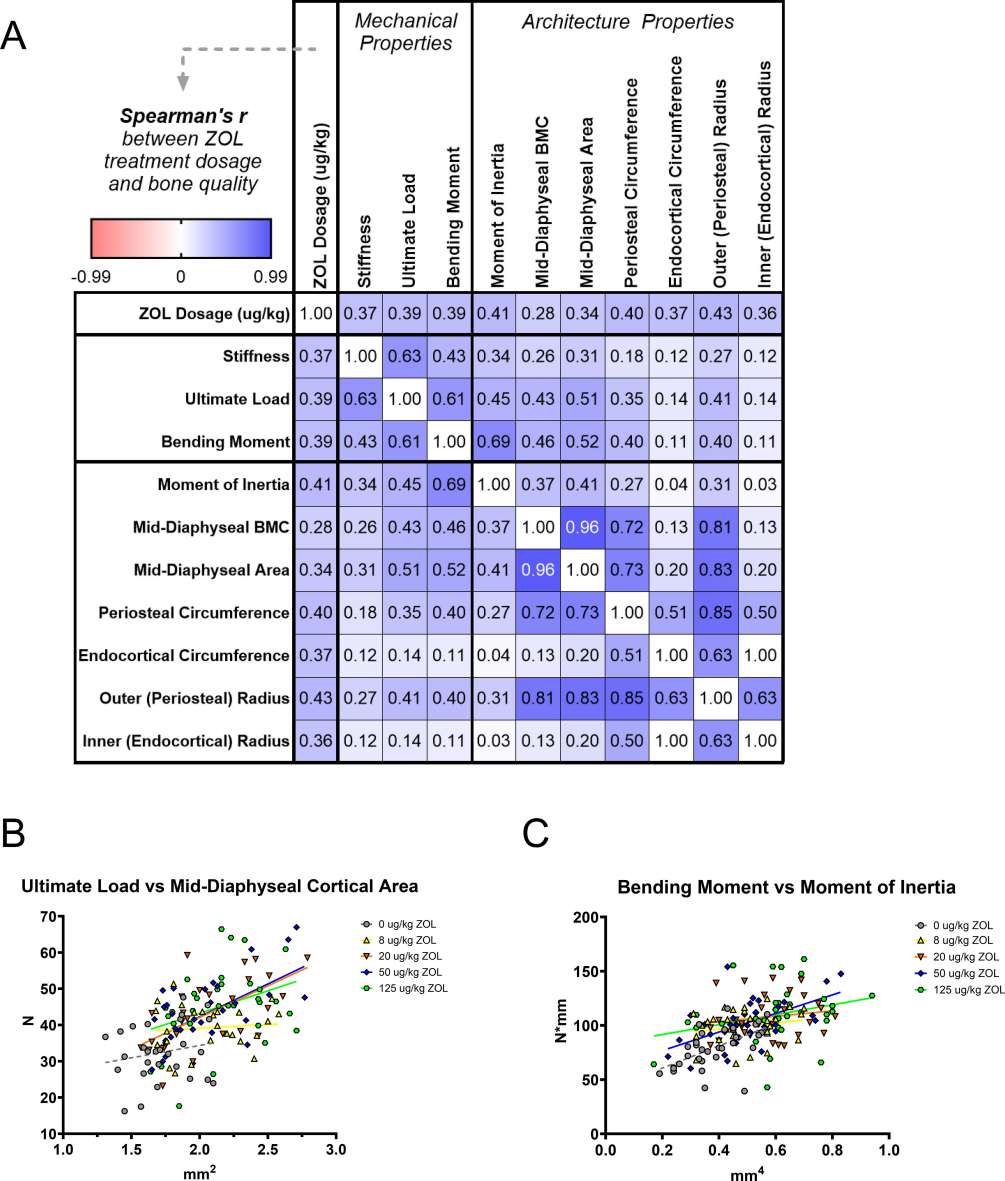
Correlation matrices and regression analyses of biomechanical and pQCT architectural bone properties. **(A)** Correlation matrices illustrate the strength and direction of linear relationships between ZOL dose, mechanical properties, and pQCT architectural variables. ZOL dose was weakly correlated with both mechanical and architectural bone quality metrics. **(B, C)** Single linear regression analyses for ZOL doses showing the relationships between **(B)** ultimate load vs. mid-diaphyseal cortical area (*r* = 0.51) and **(C)** bending moment vs. moment of inertia (r = 0.69). Statistical analyses were performed using Spearman’s correlation with two-tailed tests to determine *p*-values. Interpretation of Spearman’s correlation coefficient (R) followed a previously published classification (50): 0.90–1.00 (very high), 0.70–0.90 (high), 0.50–0.70 (moderate), 0.30–0.50 (low), and 0.00–0.30 (negligible).

Despite the significant effects of ZOL on mid-diaphyseal structural and biomechanical properties (**Figures 2**, **3**), *Spearman’s* correlation coefficients (*r*) revealed only weak associations between ZOL dose and specific biomechanical (stiffness, ultimate load and bending moment) and diaphyseal architectural properties (moment of inertia, mid-diaphyseal BMC, area, periosteal and circumferences, and outer and inner radii) (**Figure 4**). Furthermore, correlations between biomechanical and architectural properties were calculated and categorized according to established criteria (50). Relationship strengths were classified as negligible (0.00-0.30), low (0.30-50) or moderate (0.50-0-70) (**Figure 4**). We conducted single linear regression analyses to understand the dose-dependent roles in these associations better. In **Figures 4B, C**, we depicted ultimate load vs. cortical area (r = 0.51) and bending moment vs. moment of inertia (r = 0.69) as examples. The analysis comparing cortical area and ultimate load across various ZOL doses revealed a dose-dependent relationship between the two variables. Specifically, in the VEH (P = 0.32) and osteoporosis dose of 8𝜇g/kg (P = 0.66) doses, no significant linear relationship was detected. In contrast, significant associations were observed at both the 20𝜇g/kg (P = 0.0004) and 50𝜇g/kg (P = 0.0004) doses, indicating that at these levels, cortical area predicts ultimate load. The highest dose, 125𝜇g/kg, demonstrated a trend for statistical significance (P = 0.053). Single linear regression analysis of the relationship between bending moment and moment of inertia across different ZOL doses revealed a varied pattern of significance. A strong, significant association was evident in both the VEH (P = 0.0006) and the 50 𝜇g/kg group (P = 0.0006), indicating that the moment of inertia reliably predicted the bending moment at these doses. However, this relationship was not statistically significant at the 8𝜇g/kg (P = 0.29) 20𝜇g/kg (P = 0.394) or 125𝜇g/kg (P = 0.087) dose levels.

### Study 2

#### Age-related femoral pQCT and biomechanical variables in VEH-treated rats

**Figure 5:**
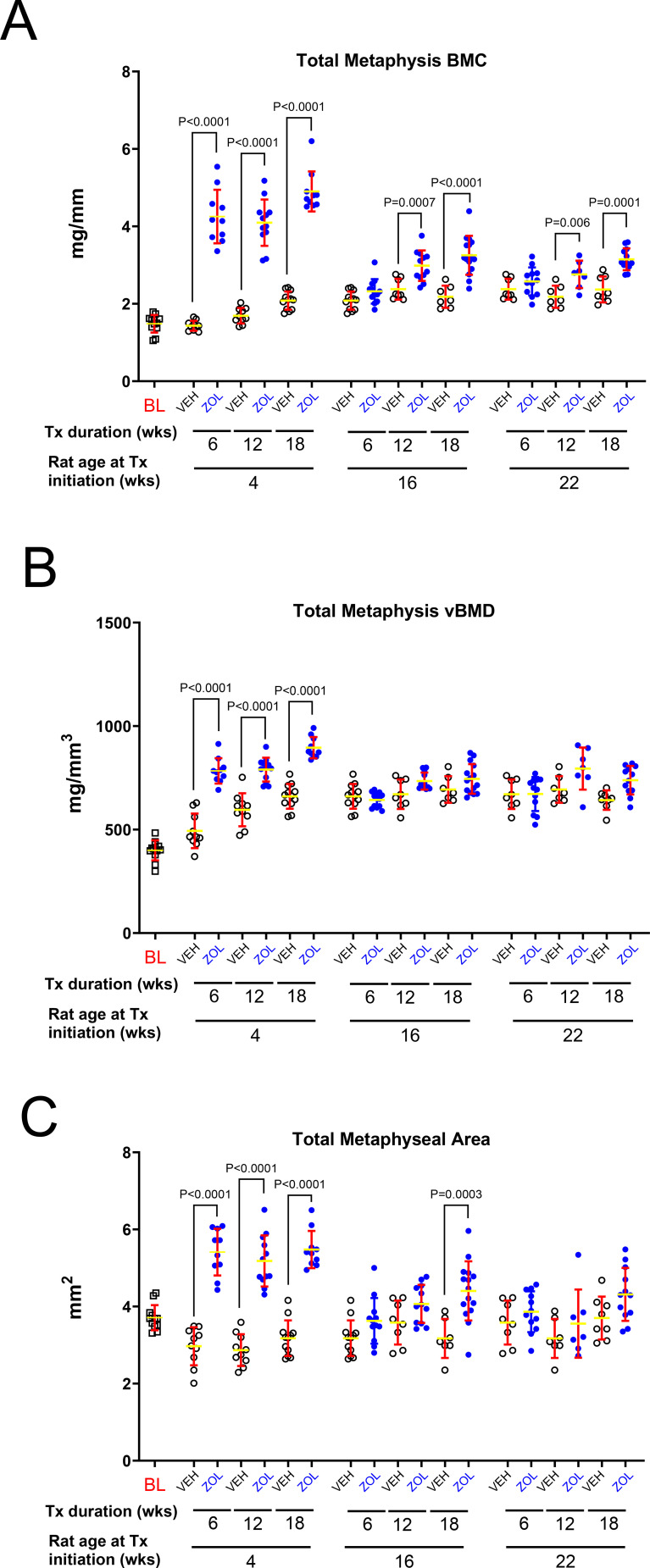
pQCT measurements at the distal femoral metaphysis in rice rats treated with an oncologic dose of zoledronic acid (ZOL) starting at different ages and applied for different durations. Rats received ZOL 80 µg/kg IV (tail vein) every 4 weeks, starting at 4, 16, or 22 weeks of age (infantile/juvenile, young adult, mature adult), and were treated for 6, 12, or 18 weeks. **(A)** total metaphysis bone mineral content (BMC), **(B)** total metaphysis volumetric bone mineral density (vBMD), and **(C)** total metaphyseal area. Individual values are plotted as open circles for VEH-treated rats and blue circles for ZOL-treated rats, with bars indicating means ± SD. Baseline (BL): 4 weeks. Welch’s t-test was used to compare each variable between the VEH and ZOL groups at each age. To mitigate the overall Type 1 error rate across multiple comparisons, a stringent significance threshold of P ≤ 0.01 was adopted.

**Figure 6:**
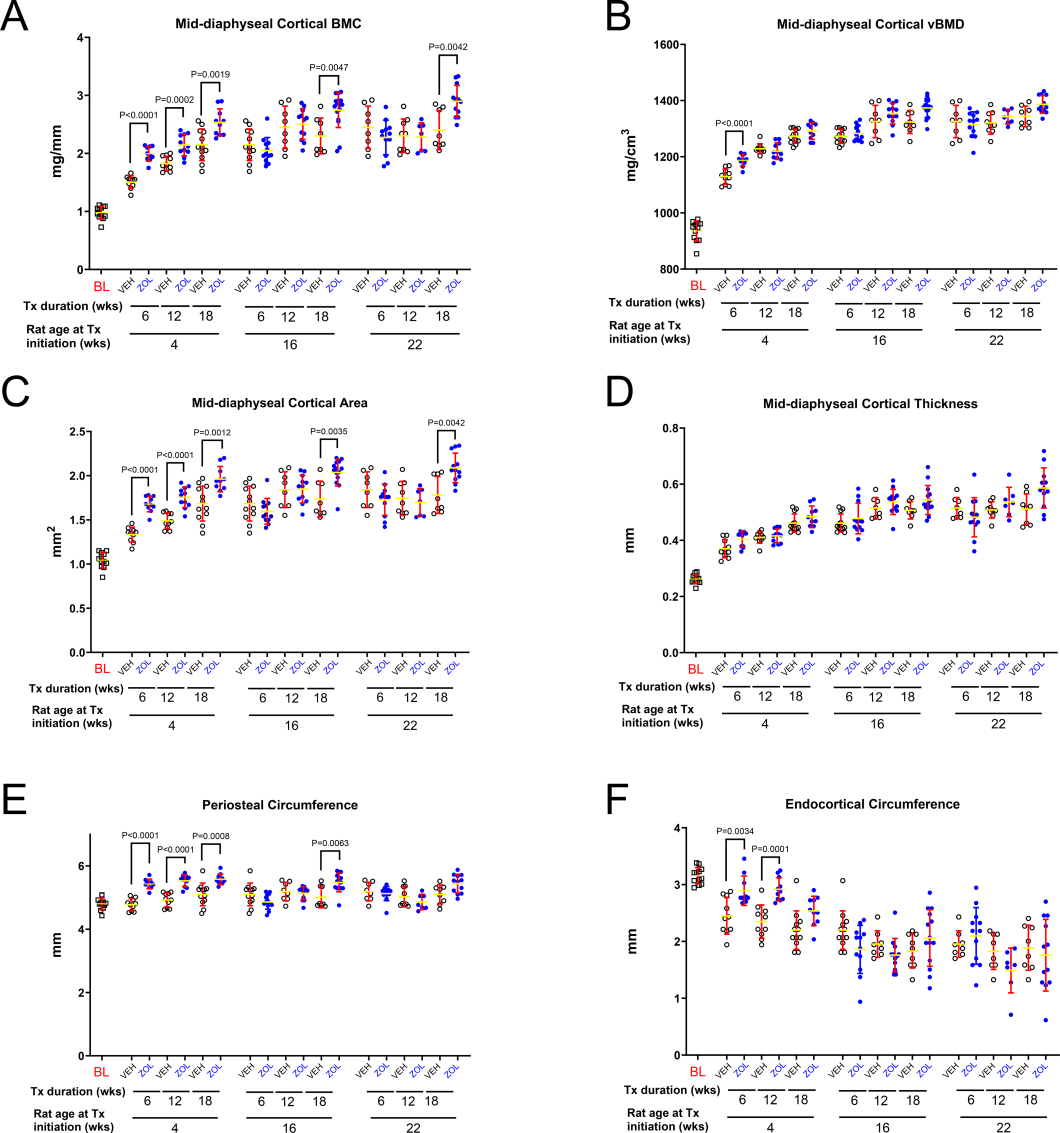
pQCT measurements at the femoral mid-diaphysis in rice rats treated with an oncologic dose of zoledronic acid (ZOL) starting at different ages and for different durations. Rats received ZOL 80 µg/kg or saline (VEH) IV (tail vein) every 4 weeks, starting at 4, 16, or 22 weeks of age (infantile/juvenile, young adult, mature adult), and were treated for 6, 12, or 18 weeks. **(A)** mid-diaphyseal cortical bone mineral content (BMC), **(B)** cortical volumetric BMD (vBMD), **(C)** mid-diaphyseal cortical area, **(D)** mid-diaphyseal cortical thickness, **(E)** periosteal circumference, and **(F)** endocortical circumference. Individual values are plotted as open circles for VEH-treated rats and blue circles for ZOL-treated rats, with bars indicating means ± SD. Baseline (BL): 4 weeks. Welch’s t-test was used to compare each variable between the VEH and ZOL groups at each age. To mitigate the overall Type 1 error rate across multiple comparisons, a stringent significance threshold of P ≤ 0.01 was adopted. Brackets denote pairwise comparisons: red, VEH groups of different ages; black, ZOL dose group comparisons at each time point.

**Figure 7:**
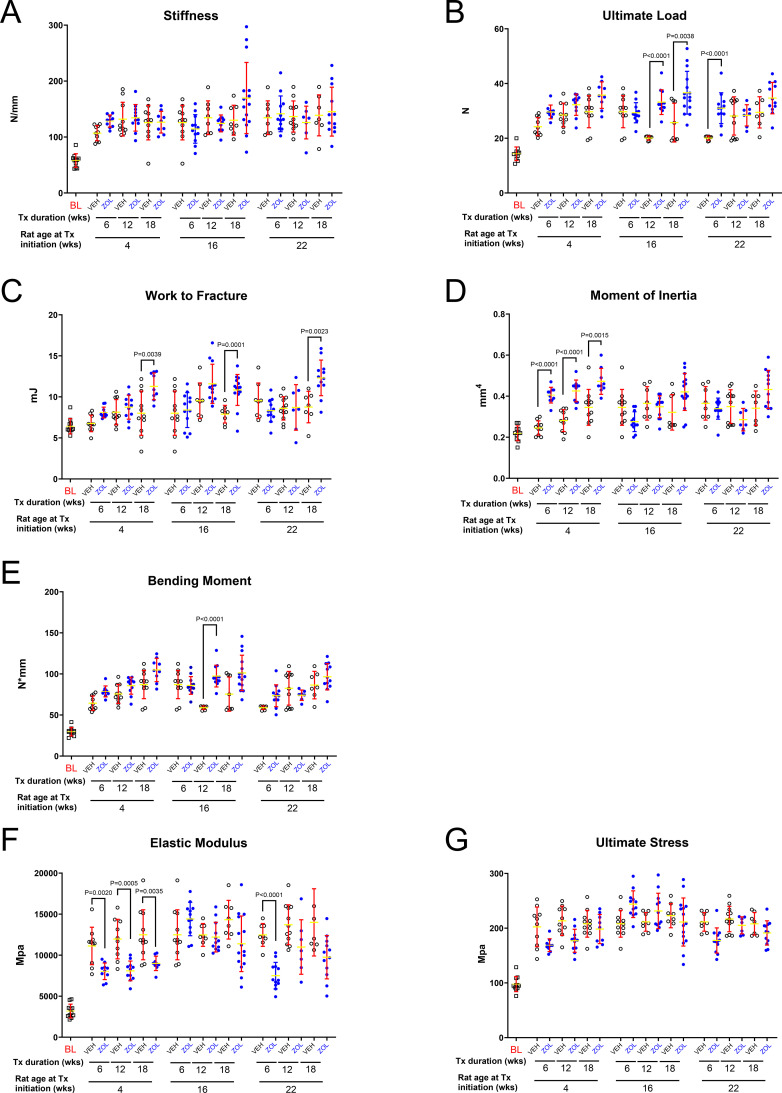
Biomechanical properties at the femoral mid-diaphysis in rice rats treated with an oncologic dose of zoledronic acid (ZOL) starting at different ages and for different durations. Rats (n = 10–15/group) received ZOL 80 µg/kg or saline (VEH) IV (tail vein) every 4 weeks, starting at ages 4, 16, or 22 weeks (infantile/juvenile, young adult, mature adult) and treated for 6, 12, or 18 weeks. **(A)** stiffness (N/mm), **(B)** Ultimate load (N/mm), **(C)** work to fracture (mJ); **(D)** moment of inertia (mm^4^), **(E)** bending moment (N*mm), **(F)** elastic modulus (MPa), and **(G)** ultimate stress (MPa). Individual values are plotted as open circles for VEH-treated rats and blue circles for ZOL-treated rats, with bars indicating means ± SD. Baseline (BL): 4 weeks. Welch’s t-test was used to compare each variable between the VEH and ZOL groups at each age. To mitigate the overall Type 1 error rate across multiple comparisons, a stringent significance threshold of P ≤ 0.01 was adopted. Brackets denote pairwise comparisons: red, VEH groups of different ages; black, ZOL dose group comparisons at each time point.

As in other rodent species and in Study 1, pQCT and biomechanical variables in rice rats are influenced by age (48). To evaluate the effects of ZOL on bone growth and development, we first present separately cancellous and cortical pQCT and biomechanical data from VEH rats to clearly visualize effects attributable only to age in **Supplementary Figures 2**-**4**. In **Figures 5**–**7**, VEH-treated rats serve as controls for each corresponding ZOL treatment condition.

#### Distal femoral metaphyseal pQCT variables in VEH rats from infantile/juvenile to mature adulthood

Distal femoral metaphyseal BMC was stable from ages 4 to 16 weeks but was significantly higher at ages 22–40 weeks, where it reached a new plateau (**Supplementary Figure 2A**). Total metaphyseal vBMD increased during ages 4 to 16 weeks, then was stable through age 40 weeks (**Supplementary Figure 2B**). No significant differences were observed in total metaphyseal area over time (**Supplementary Figure 2C**).

#### Mid-femoral diaphyseal cortical pQCT variables in VEH rats

We observed a progressive increase in femoral mid-diaphyseal BMC and area from age 4 weeks to age 22 weeks, after which cortical BMC and area values plateaued (**Supplementary Figures 3A, C**). Similarly, cortical vBMD and cortical thickness both showed steady increases, which plateaued by age 28 weeks (**Supplementary Figures 3B, D**). While periosteal circumference remained relatively stable (**Supplementary Figure 3E**), endocortical circumference decreased significantly and progressively until age 28 weeks (**Supplementary Figure 3F**), then plateaued through age 40 weeks.

#### Mid-femoral diaphyseal biomechanical variables in VEH rats

We observed significant increases in stiffness, ultimate load, bending moment, elastic modulus, and ultimate stress from 4 to 10 weeks of age (**Supplementary Figures 4A, B, E–G**). After age 10 weeks, these properties remained relatively stable through age 40 weeks. The moment of inertia was significantly greater at age 16 weeks than at ages 4 and 10 weeks and remained stable through age 40 weeks.

#### Distal femoral metaphysis pQCT variables in ZOL rats

when the 80 μg/kg ZOL oncologic dose was administered to 4-week-old rats, independent of treatment duration (6, 12 or 18 weeks), marked significant increases were observed in total metaphyseal BMC, vBMD, and area compared to VEH rats (**Figures 5A–C**). The magnitude of these increases did not differ significantly among the treatment-duration subgroups. When treatment was initiated in 16-week-old rats, ZOL increased cortical BMC only in the longer-duration subgroups (12 and/or 18 weeks) (**Figure 5A**), and cortical area only after 18 weeks (**Figure 5C**). When ZOL was initiated at 22 weeks of age, total BMC increased in rats treated for 12 or 18 weeks. No other variables showed significant differences across the treatment duration groups (**Figure 5**).

#### Mid-femoral diaphyseal cortical pQCT variables

In general, the most pronounced effects on BMC, cortical area, and periosteal circumference were observed when rats began treatment at age 4 weeks. When ZOL was initiated at age 4 weeks, cortical BMC, cortical area, and periosteal circumference increased after all treatment durations (**Figures 6A, C, E**). When begun in 16-week-old rats, ZOL increased cortical BMC, area, and periosteal circumference only after 18 weeks of treatment (**Figures 6A, C, E**). When treatment began at 22 weeks of age, ZOL increased cortical BMC and cortical area only with the longest treatment duration (**Figures 6A, C**).

#### Mid-femoral diaphyseal biomechanical variables

There were no differences in stiffness between ZOL-treated and VEH-treated rats, regardless of duration or age at treatment initiation (**Figure 7A**). ZOL treatment beginning at 16 weeks of age increased ultimate load after 12 and 18 weeks of treatment, and when initiated at 18 weeks of age, increased ultimate load after 6 weeks (**Figure 7B**). ZOL increased work to fracture at the femoral midshaft only when treatments lasted 18 weeks, regardless of the age at which treatment started (**Figure 7C**). The moment of inertia increased when treatment started at 4 weeks of age and continued for 6, 12, or 18 weeks, but this was not observed when treatment started in older rats (**Figure 7D**). In addition, increased bending moment was observed only when ZOL treatment started at 12 weeks of age and lasted 12 weeks (**Figure 7E**). ZOL also reduced the elastic modulus when treatment began at 4 weeks of age and continued for 6, 12, or 18 weeks, and when treatment began at 16 weeks of age and continued for 6 weeks (**Figure 7F**). Finally, there was no difference in ultimate stress between ZOL-treated and VEH-treated rats, regardless of treatment duration or age at the start of treatment (**Figure 7G**).

## Discussion

This study investigated how ZOL dose, treatment duration, and age at treatment initiation affect femoral architecture and biomechanical properties during skeletal development and maturation in female rice rats. Two complementary studies were performed (1): a dose–duration study initiating ZOL in juvenile rats and (2) an age-at-initiation study utilizing an oncologic ZOL dose, with follow-up extending into mature adulthood.

Our study exclusively used female rice rats. It is well established that sex influences bone growth patterns and architectural development. While our previous work in rice rats indicates sex-specific differences in femoral geometry and mechanical properties (48), the absence of male subjects thus represents a limitation of this study.

To distinguish ZOL-induced effects from natural aging physiological changes, we first characterized age-related skeletal changes in VEH-treated female rice rats. Four-week-old rats are in a rapid growth phase, transitioning from juveniles to adults. In these VEH rats, femoral metaphyseal vBMD typically increased, and BMC stabilized by young adulthood. In contrast, femoral mid-diaphyseal cortical vBMD, area, and thickness continued to increase steadily through young and middle adulthood, a process largely attributable to periosteal bone apposition and a decrease in endocortical circumference during early growth (51–53). Concurrently, femoral mid-diaphyseal cortical biomechanical properties, including stiffness, ultimate load, and bending moment, showed age-related increases through middle adulthood, indicating enhanced strength and resistance to fracture, accompanied by improved tissue-level properties (e.g., elastic modulus and ultimate load) (54, 55). These VEH control data are presented in **Supplementary Figures 2**–**4** and are featured in comparative displays with ZOL groups in **Figures 5**–**7**.

While femoral metaphyseal and cortical pQCT and biomechanical properties of Study 2 VEH rats were largely consistent with those of Study 1 VEH rats, some distinctions were observed: 1) total metaphyseal BMC in Study 2 VEH rats increased at a later age; 2) periosteal circumference remained almost unchanged from 4 to 40 weeks in Study 2, contrasting with the age-related increases in Study 1 VEH rats; 3) stiffness and ultimate load in Study 2 showed an early increase followed by a plateau through 40 weeks in VEH rats, unlike the more gradual increase observed in Study 1 VEH rats up to 34 weeks; and 4) work to fracture remained almost unchanged from 4 to 40 weeks in Study 2 VEH rats, contrasting with the age-related increases in Study 1 VEH rats. This variability is likely attributable to the inherent genetic diversity of *Oryzomys palustris*, an outbred species with higher heterozygosity and allelic variation than standard inbred laboratory rodents. Although unexpected, these differences establish a crucial baseline for healthy skeletal maturation in rice rats and underscore the importance of accounting for an animal’s specific age and developmental stage when evaluating ZOL’s effects on bone health, without altering the overall interpretation of our studies.

The effects of ZOL on cancellous and cortical bone structure and cortical biomechanical properties were predominantly modulated by treatment regimen factors: dose levels, duration, and age at initiation. ZOL consistently increased total metaphyseal BMC, vBMD, and cross-sectional area in juvenile rats (4 weeks of age). Moreover, oncologic doses (20 and 125 µg/kg) typically resulted in more pronounced gains in these parameters than the 8 µg/kg osteoporosis dose.

Although treatment duration alone did not uniformly change the degree of the metaphyseal response, longer exposures (≥18 weeks) accentuated the differences between ZOL’s oncologic and osteoporosis doses. These results indicate that the distal metaphysis of the growing femur, as an integral representation of the cancellous bone compartment of the appendicular skeleton, is very responsive to antiresorptive therapy and that higher doses of ZOL produce a greater increase in cancellous bone mass and density than the low-dose of ZOL. Furthermore, ZOL induced duration-dependent changes in cortical bone pQCT parameters in young (4-week-old) rice rats. After 12–18 weeks, ZOL increased both the mid-diaphyseal cortical area and endocortical circumference. After 24 weeks, nearly all ZOL doses led to significant increases in cortical BMC, area and periosteal circumference. The oncologic doses, however, tend to induce greater increases in these variables than the osteoporosis dose. Importantly, ZOL did not affect cortical vBMD or cortical thickness at any dose level or duration when ZOL was initiated in rats at 4 weeks of age. Overall, ZOL tended to increase cortical area by allowing periosteal expansion to continue unabated, particularly after 24 weeks of treatment, without altering cortical bone density or thickness. Our findings are consistent with the juvenile mouse study by Bartlow et al. (56), which reported a sustained increase in cortical area due to periosteal apposition in ZOL-treated mice from 4 to 28 weeks of age. However, that study also observed a concomitant decline in cortical tissue mineral density and a modest increase in cortical thickness that emerged early in the treatment course. In another study, N-BPs given to growing mice enhanced bone quantity: ZOL and alendronate increased cortical area and thickness, while all N-BPs increased trabecular bone volume and number. However, these changes were accompanied by reduced cortical tissue mineral density and blunted material-level strength at later time points, without impairing longitudinal bone growth (21).

Initiating ZOL treatment at 4 weeks of age (infant/juvenile rats) produced significant effects on femoral cortical biomechanical properties. After 12 weeks of ZOL, stiffness was significantly higher at all doses (8–125 µg/kg) compared with VEH controls, with no other biomechanical parameters showing significant differences. With longer ZOL exposures (18–24 weeks), significant increases were observed across several ZOL dose levels for stiffness, ultimate load, moment of inertia, and bending moment. At the longest exposure (30 weeks), the response to ZOL became more nuanced, with a significant effect on the moment of inertia only. Despite ZOL inducing significant increases in these biomechanical parameters, dose-dependent effects were rarely observed, particularly between 12 and 18 weeks of treatment. It was only after 24 weeks of treatment that we observed a significant increase at 20, 50, and/or 125 µg/kg relative to the osteoporosis dose (8 µg/kg), and this was observed only in ultimate load, moment of inertia, and bending moment. This indicates that while the osteoporosis dose of ZOL enhances bone strength without marked differences compared with higher doses, longer exposure to mid-range oncologic doses may confer additional biomechanical benefits and fracture resistance. Supporting the relevance of treatment duration and intensity in a distinct physiological setting, a study in adult ovariectomized female rats (rather than young intact females) showed that the magnitude of ZOL’s effects on bone depended on both dose and time over a year-long treatment regimen (22).

In study 1, we also showed that ZOL increased stiffness and ultimate load, which are associated with increased moment of inertia driven by bone external size (periosteal circumference) and BMC. The increase in structure-level biomechanical properties, alongside unchanged tissue-level variables (elastic modulus, ultimate stress), suggests that ZOL’s effects are driven by bone quantity rather than bone quality. While ZOL improved bone strength in growing rice rats, these enhancements did not follow a consistent dose-dependent pattern. Indeed, although ZOL significantly increased many mid-diaphyseal structural and biomechanical properties, these benefits often did not increase linearly with dose. The weak Spearman correlations between ZOL dose levels and architectural and biomechanical outcomes suggest a plateau in efficacy, or a “ceiling effect.” This indicates that the maximum effects were often achieved at lower doses, with no significant further increases at the oncologic doses. Consequently, the relationship between ZOL and bone strength appears nonlinear, with doses above a threshold yielding diminishing returns for both parameters. Thus, the lack of a consistent, dose-dependent effect of ZOL implies that careful consideration must be given to the treatment duration and the specific biomechanical property one aims to improve.

In Study 2, we examined how age at ZOL treatment initiation influences both pQCT variables at the distal femoral metaphysis and mid-diaphysis and biomechanical properties at the mid-diaphysis. After administering oncologic doses (80 µg/kg/q4 wks) of ZOL to 4-week-old rats, we observed significant increases in total metaphyseal BMC, vBMD, and area compared with VEH rats, regardless of treatment duration (6, 12, or 18 weeks). However, when treatments began at 16 or 22 weeks of age, ZOL increased metaphyseal BMC and area only with longer treatment durations. Likewise, ZOL induced changes in cortical bone pQCT parameters, with effects varying by age at treatment initiation and treatment duration. Furthermore, in 4-week-old rats given oncologic doses of ZOL, cortical BMC, area, and periosteal and endocortical circumferences increased significantly, largely independent of treatment duration (6, 12, or 18 weeks).

In contrast, when treatment began at ages 16 or 22 weeks, increases in cortical BMC, area, and periosteal circumference appeared only with longer treatment duration, with minimal effects on cortical vBMD and thickness. These results suggest that bone responsiveness to ZOL declines with maturation, requiring longer exposures in older rats to achieve detectable changes. Overall, ZOL’s effects on cancellous and cortical structure depend on duration, and critically, age at initiation. Early treatment yields more pronounced, widespread changes in both metaphyseal and cortical compartments, whereas later initiation requires longer courses to approach similar effects. These findings are consistent with ZOL’s maximal efficacy during active growth.

In Study 2, we also examined how the age at initiation of oncologic-dose ZOL affected the biomechanical properties of the femoral diaphysis. ZOL produced relatively inconsistent changes in femoral biomechanical properties, regardless of starting age or treatment duration. Work to fracture increased only with 18 weeks of ZOL, independent of age at initiation. Oncologic ZOL doses increased ultimate load at 12 and 18 weeks when treatment began at 16 and 22 weeks, respectively. The moment of inertia increased when ZOL started at age 4 weeks and continued for 6, 12, or 18 weeks, but not when treatment began later. The bending moment increased only when ZOL was initiated at 16 weeks of age and continued for 12 weeks. Notably, oncologic doses reduced elastic modulus when treatment began at age 4 weeks and lasted 6, 12 or 18 weeks, and when started at age 16 weeks and continued for 6 weeks. Our findings indicate that the effects of oncologic doses of ZOL on the biomechanical properties of the femoral midshaft are more subtle and less consistent than their effects on bone structure assessed by pQCT. In addition, while some ZOL doses improved work-to-fracture and, to a lesser extent, ultimate load and moment of inertia, these effects were highly dependent on age at treatment initiation and exposure duration. The absence of consistent increases in stiffness and ultimate stress, together with reductions in elastic modulus under certain conditions, underscores the complex, sometimes unpredictable impact of ZOL on bone biomechanical properties, particularly when administered at different stages of skeletal development.

While our study provides valuable insights into the effects of ZOL on the growing skeleton, it is important to acknowledge several limitations. First, our investigation focused exclusively on the appendicular skeleton (femur), leaving the effects on the axial skeleton unassessed. Second, the study primarily employed a descriptive rather than strictly mechanistic approach. While pQCT and biomechanical testing yielded robust structural and mechanical data, our analysis lacked detailed microarchitectural characterization (e.g., micro-CT of cancellous microarchitecture), cellular-level mechanistic verification (e.g., histomorphometry), and growth plate assessment, all of which are important for fully understanding bone development and modeling and remodeling in the growing skeleton. Furthermore, bone turnover markers were not evaluated, preventing direct confirmation of ZOL’s systemic suppression of modeling and remodeling. Third, this investigation was limited to female rice rats. This constitutes a limitation on the generalizability of our findings, particularly regarding potential sex-specific differences in ZOL response, given that sex influences bone growth and our previous work indicates relevant sexual dimorphism in this species. Fourth, the limited sample sizes (n=7–15 per subgroup) may affect statistical power and highlight the need for future targeted studies with larger groups. Fifth, the inherent variability observed between vehicle groups in Studies 1 and 2, reflecting the nature of outbred laboratory animal species, is an important consideration for future reproducibility efforts. Lastly, a direct comparison to more conventional rodent models (e.g., C57BL/6 mice, Sprague-Dawley rats, Wistar rats, or Lewis rats) was not undertaken, limiting broader inter-species translational comparisons. These identified areas represent important avenues for future research.

In conclusion, ZOL administration during skeletal development increases metaphyseal and cortical bone area. It enhances mid-diaphyseal mechanical properties in a time-dependent manner, with oncologic doses sometimes producing greater structural improvements than low-dose regimens. Furthermore, our findings not only support the potential for ZOL to improve bone strength during development but also underscore the need for careful dose selection, age-appropriate treatment planning, and rigorous long-term safety assessment before broad clinical use in pediatric populations. Future studies should include multisite, longer-term preclinical work, pharmacokinetic bridging to human exposures, and well-controlled clinical trials with systematic long-term follow-up to define optimal pediatric dosing strategies and monitor growth and skeletal health outcomes.

## Data Availability

The datasets presented in this study can be found in online repositories. The names of the repository/repositories and accession number(s) can be found in the article/**Supplementary Material**.
