# Supplementary figures and images for: Dose-, duration- and age-dependent effects of zoledronic acid on bone structure and mechanical properties in growing rice rats
Source: Front Endocrinol (Lausanne). 2026 Jun 3;17:1772372. doi: 10.3389/fendo.2026.1772372 (PMC13271957; doi:10.3389/fendo.2026.1772372)

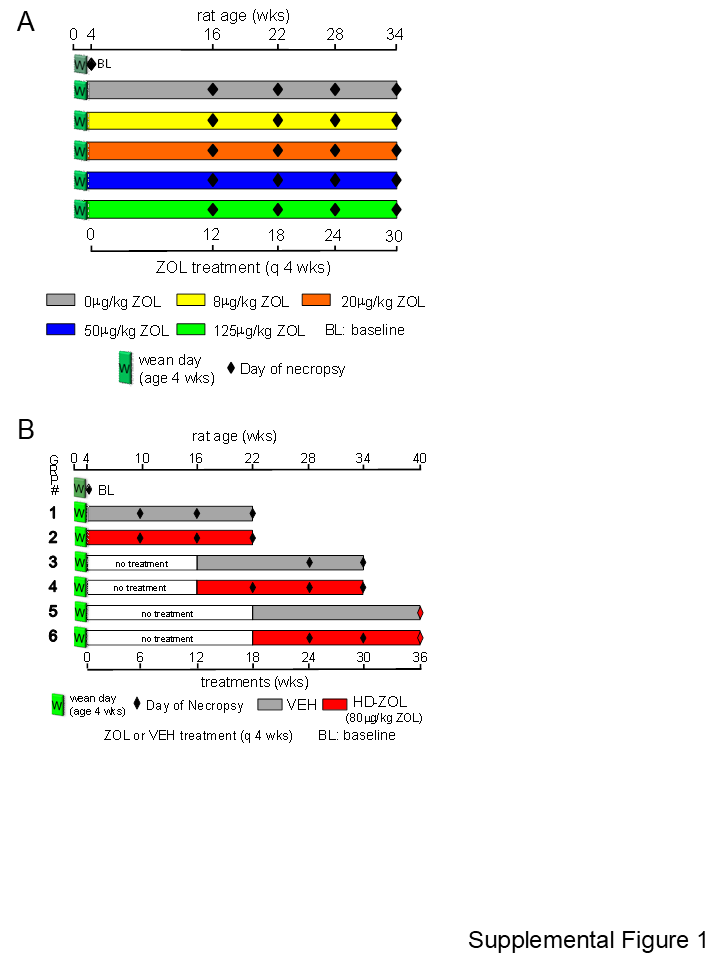

Supplement: Supplementary file 1 [file Image1.tif]

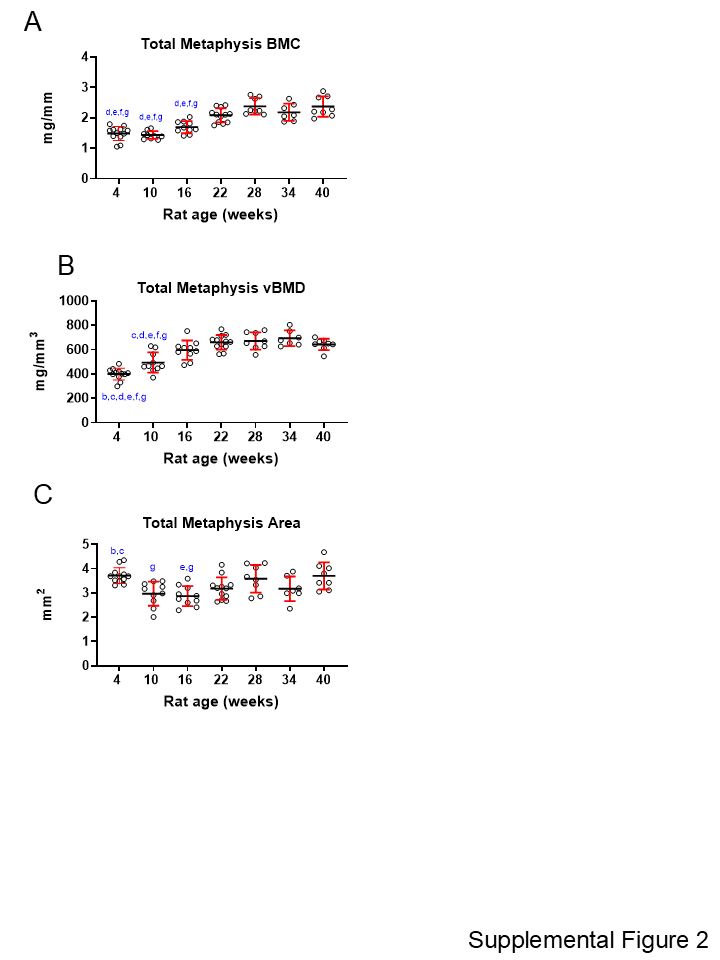

Supplement: Supplementary file 2 [file Image2.tif]

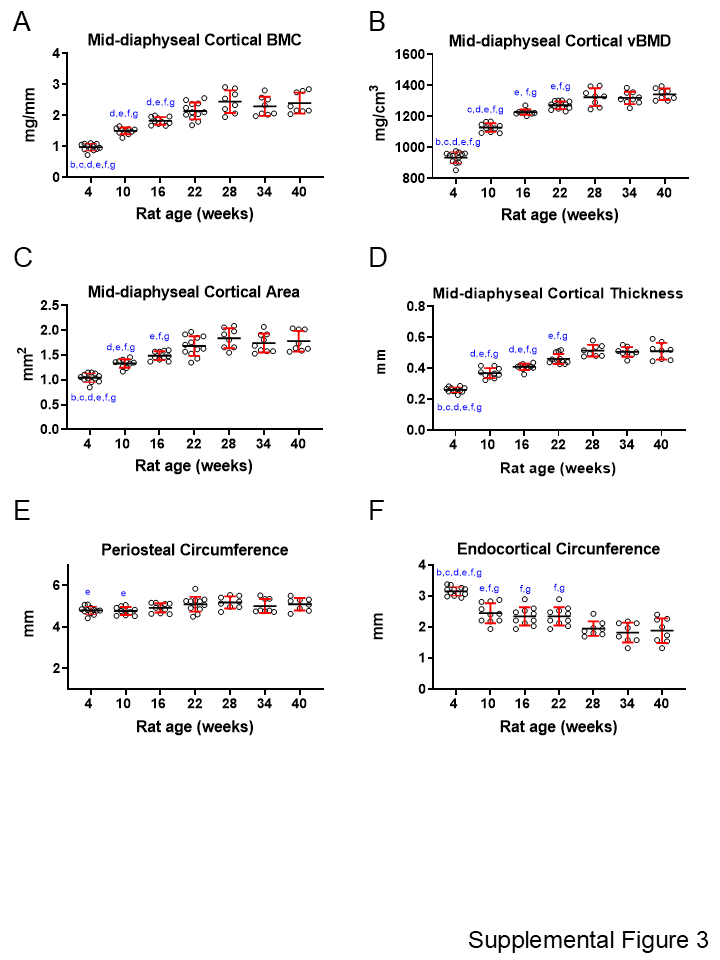

Supplement: Supplementary file 3 [file Image3.tif]

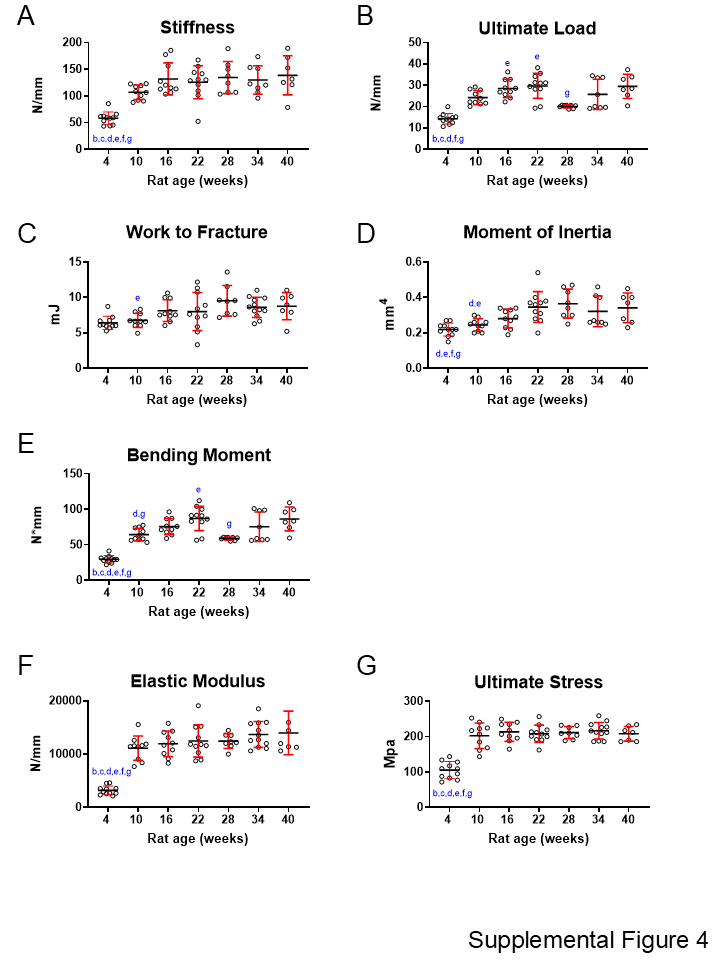

Supplement: Supplementary file 4 [file Image4.tif]

## Slide 1
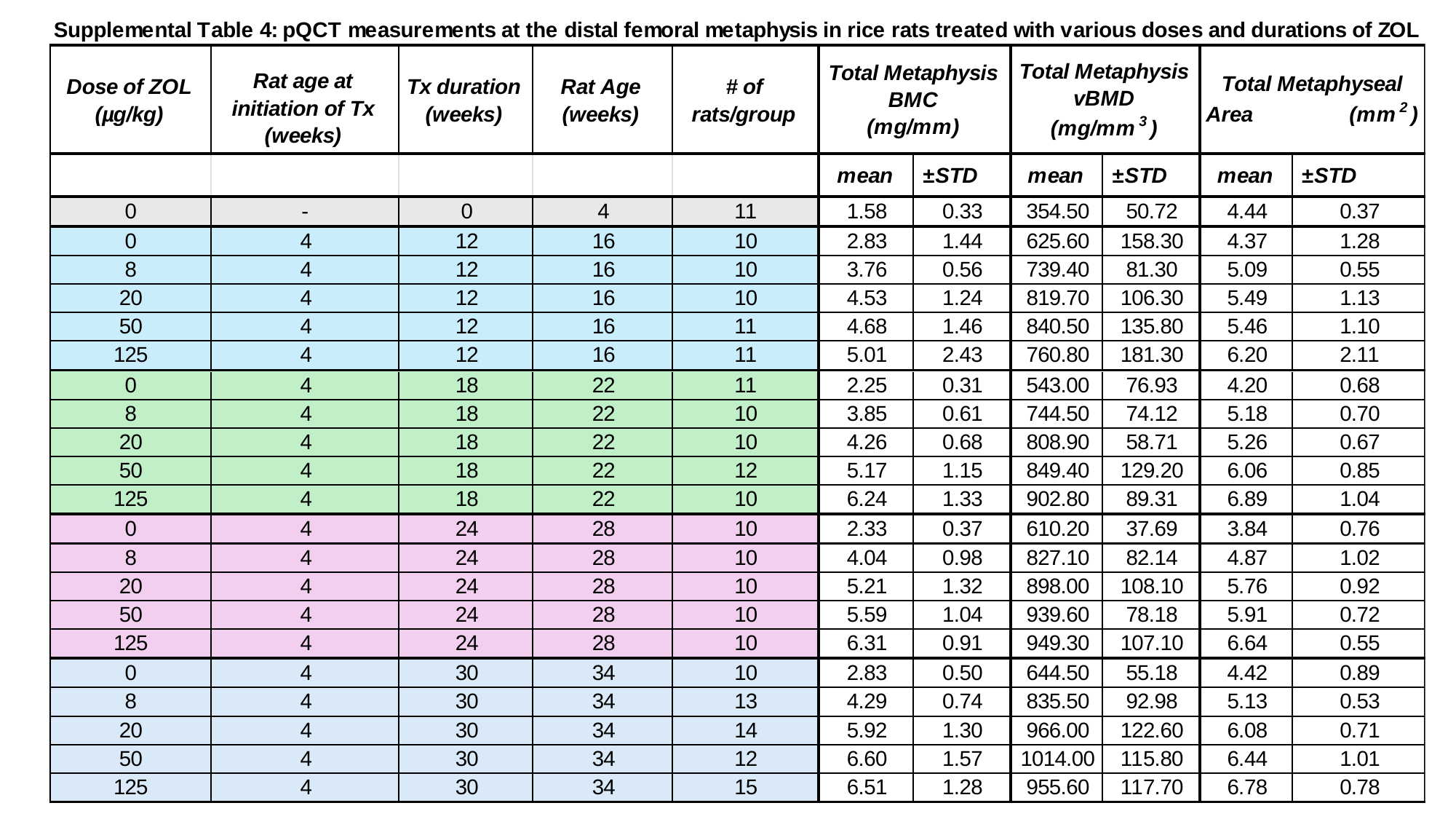

Supplement: Supplementary file 8 [file Presentation4.pptx]

## Slide 1
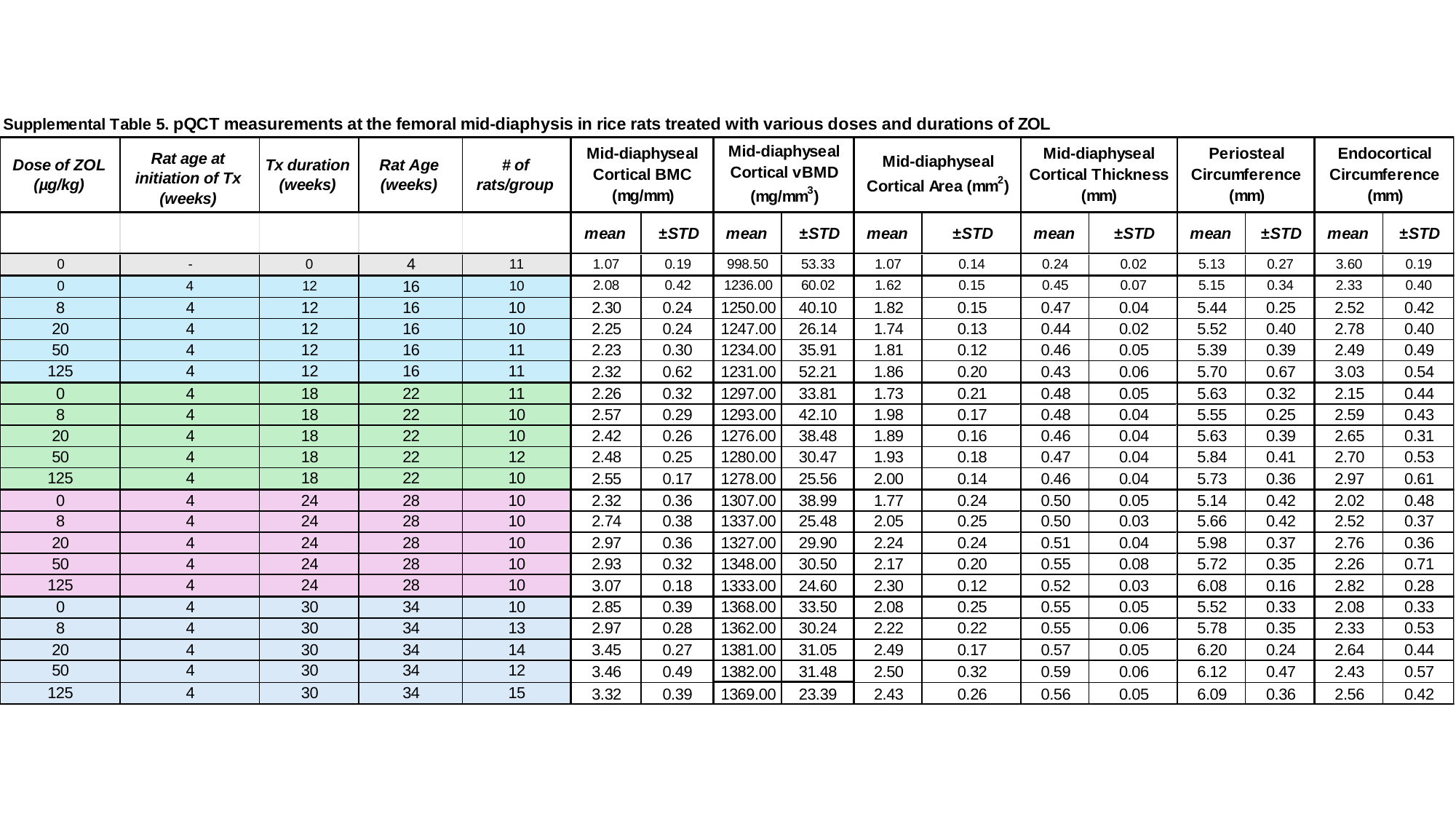

Supplement: Supplementary file 9 [file Presentation5.pptx]

## Slide 1
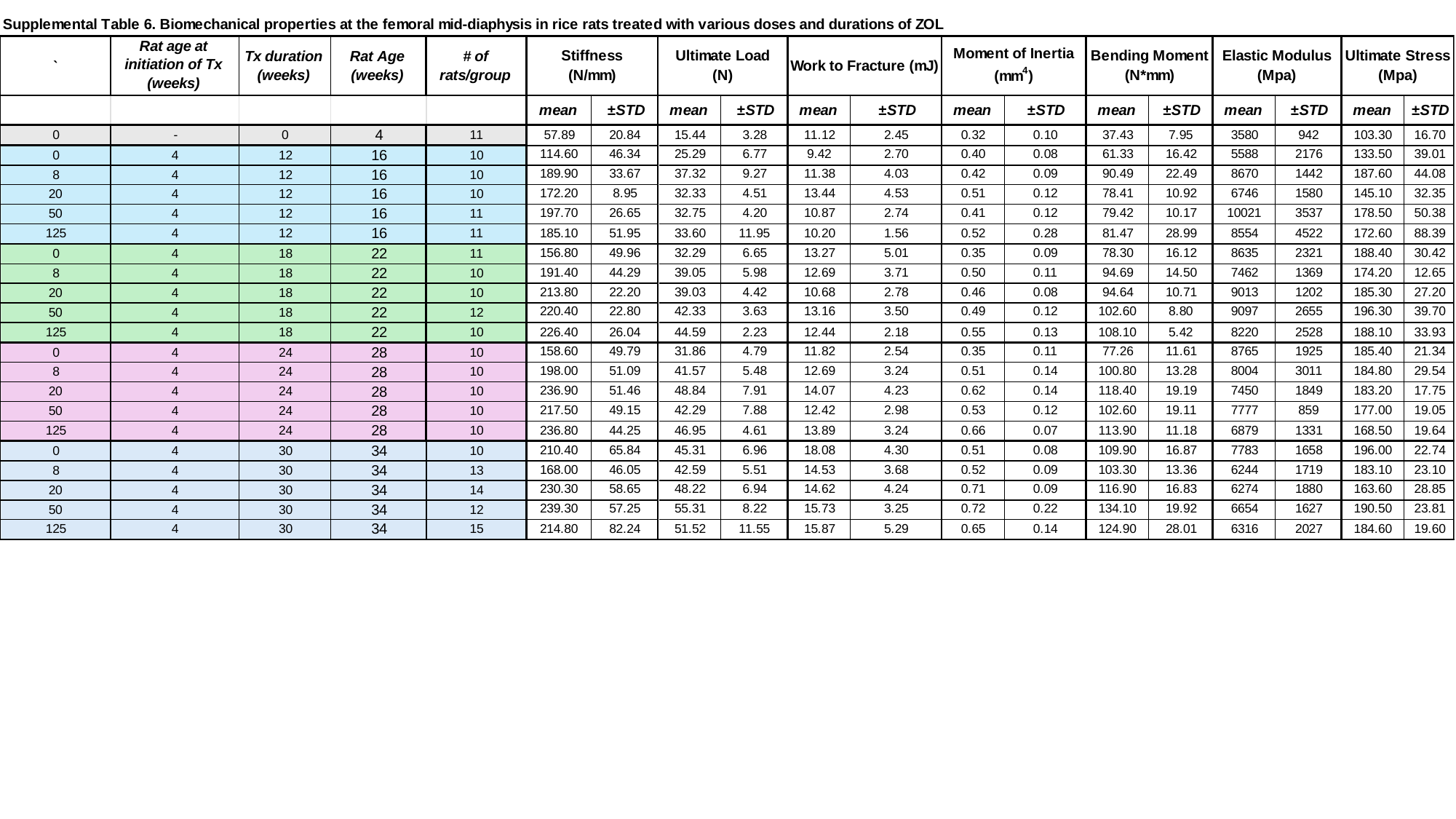

Supplement: Supplementary file 10 [file Presentation6.pptx]

## Slide 1
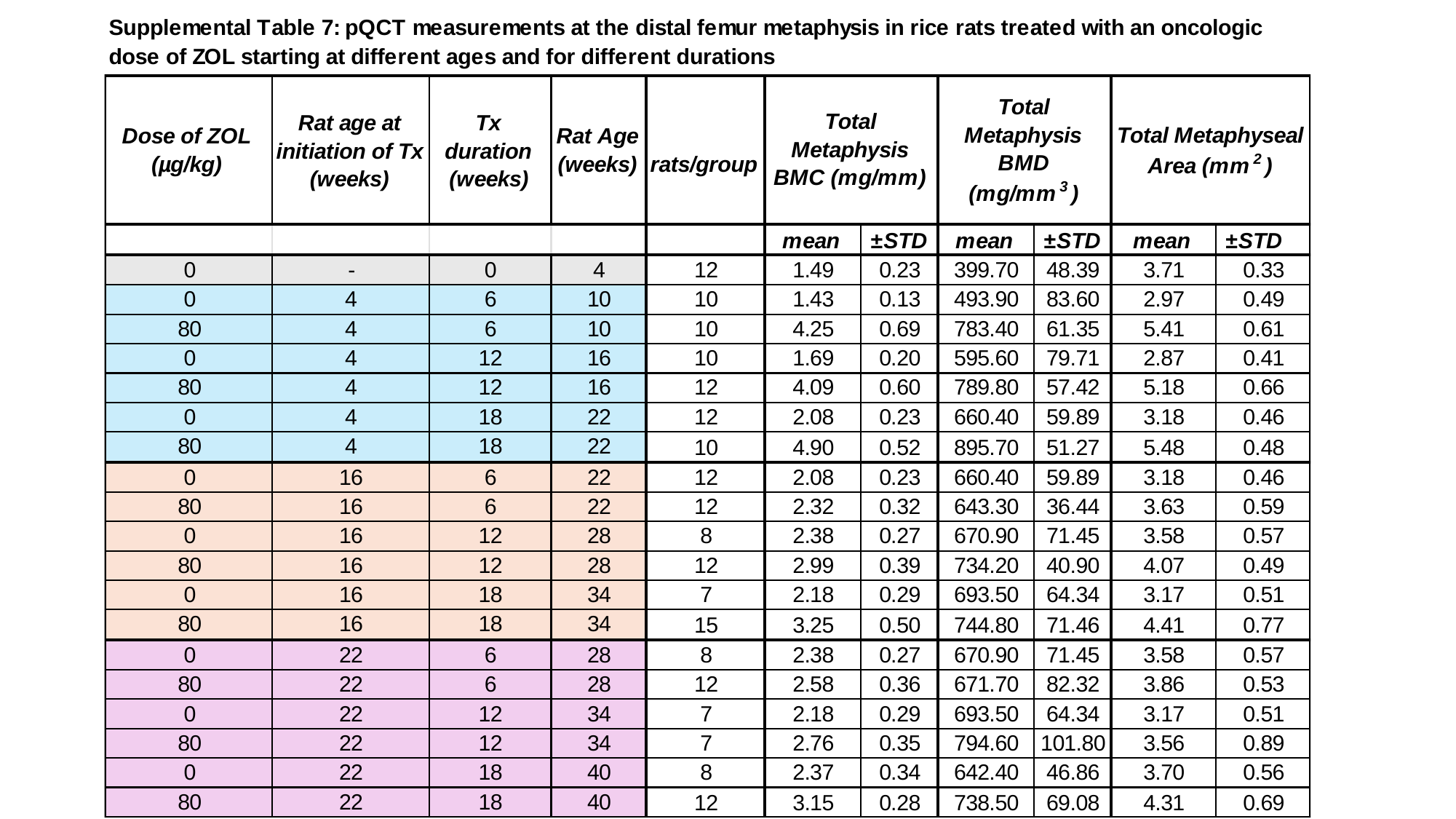

Supplement: Supplementary file 11 [file Presentation7.pptx]

## Slide 1
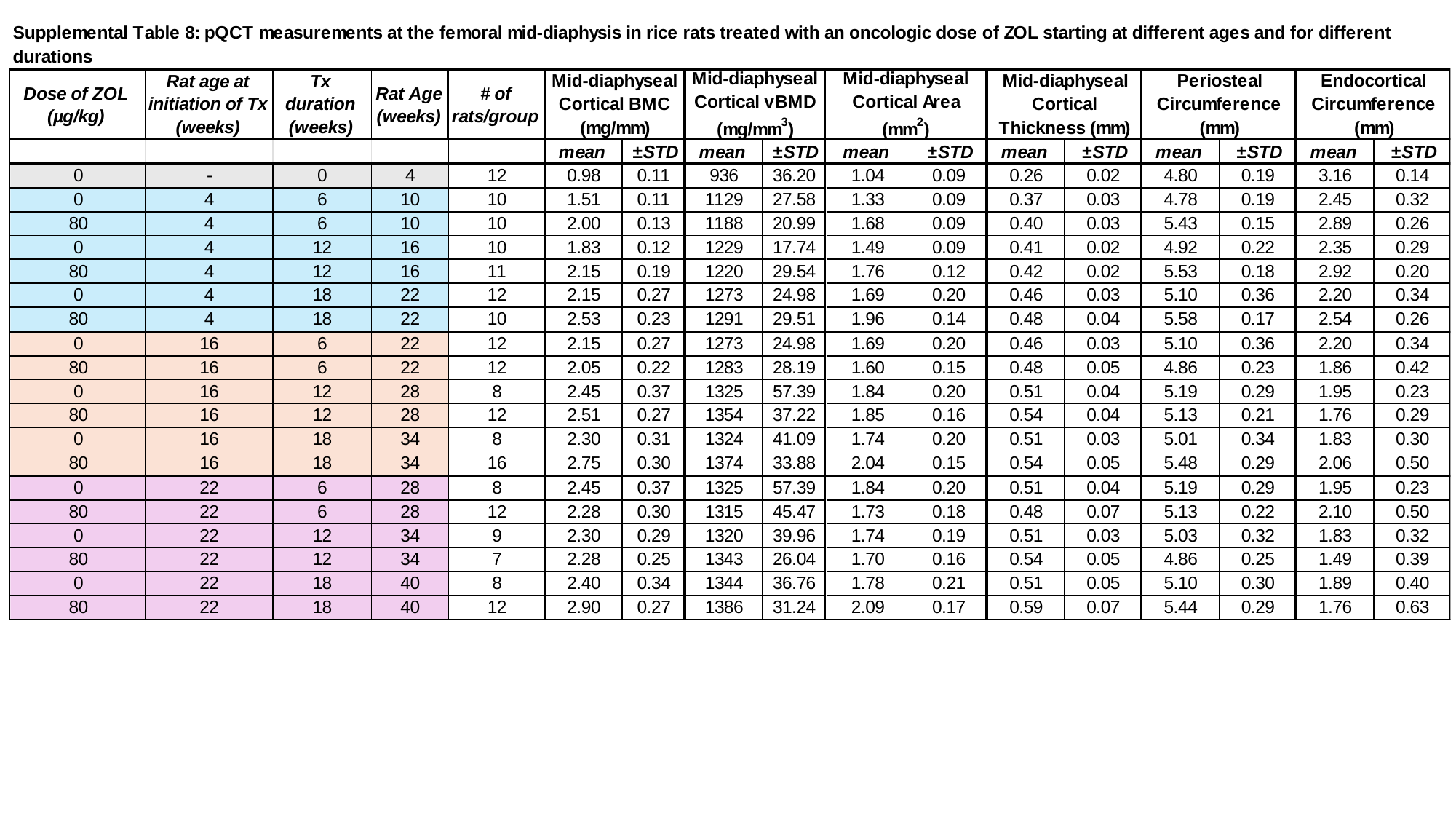

Supplement: Supplementary file 12 [file Presentation8.pptx]

## Slide 1
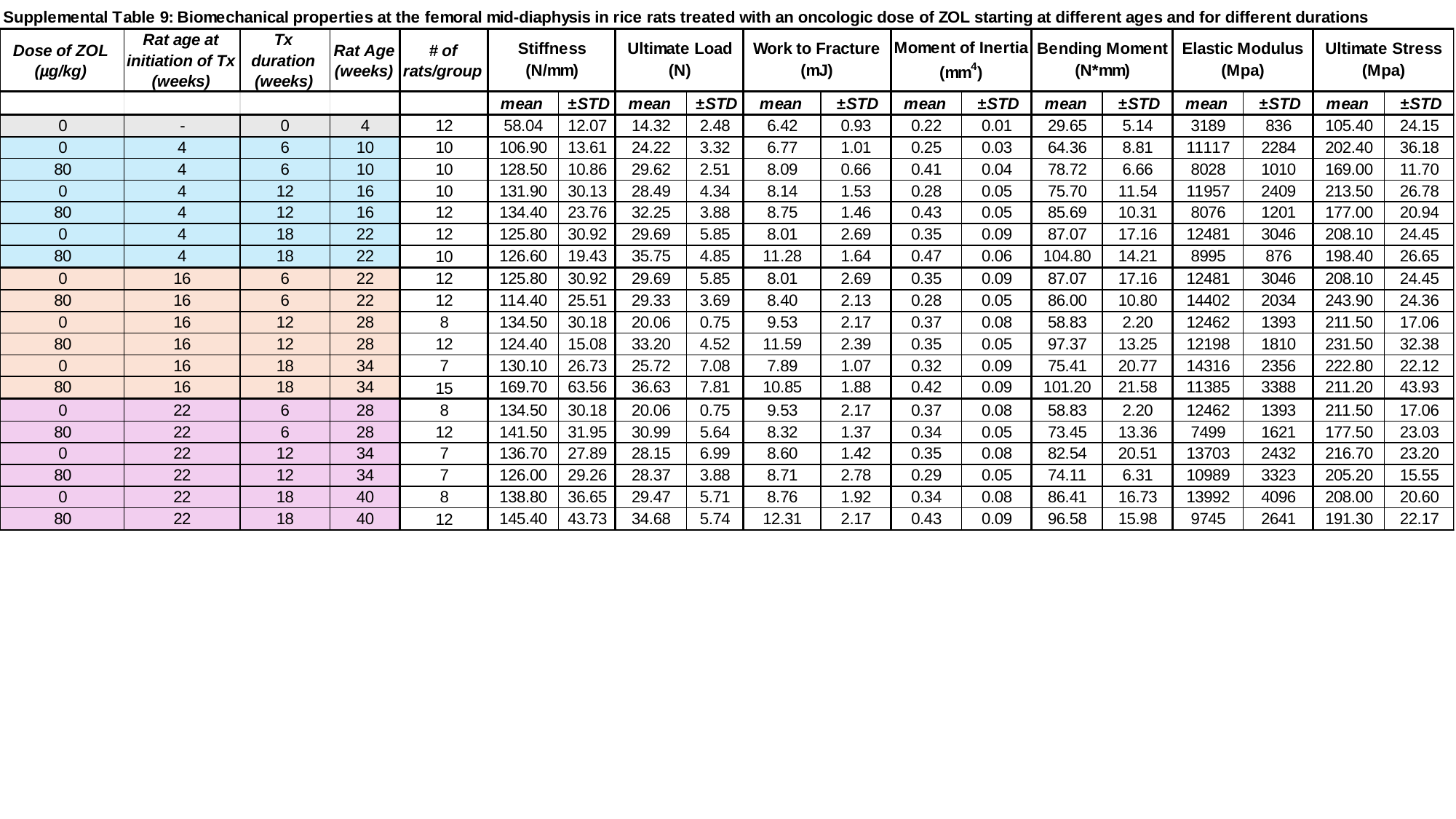

Supplement: Supplementary file 13 [file Presentation9.pptx]
