# Supplementary material for: Dose-, duration- and age-dependent effects of zoledronic acid on bone structure and mechanical properties in growing rice rats
Source: Front Endocrinol (Lausanne). 2026 Jun 3;17:1772372. doi: 10.3389/fendo.2026.1772372 (PMC13271957; doi:10.3389/fendo.2026.1772372)
Supplement: Supplementary file 5 [file Presentation1.pptx]

## Slide 1
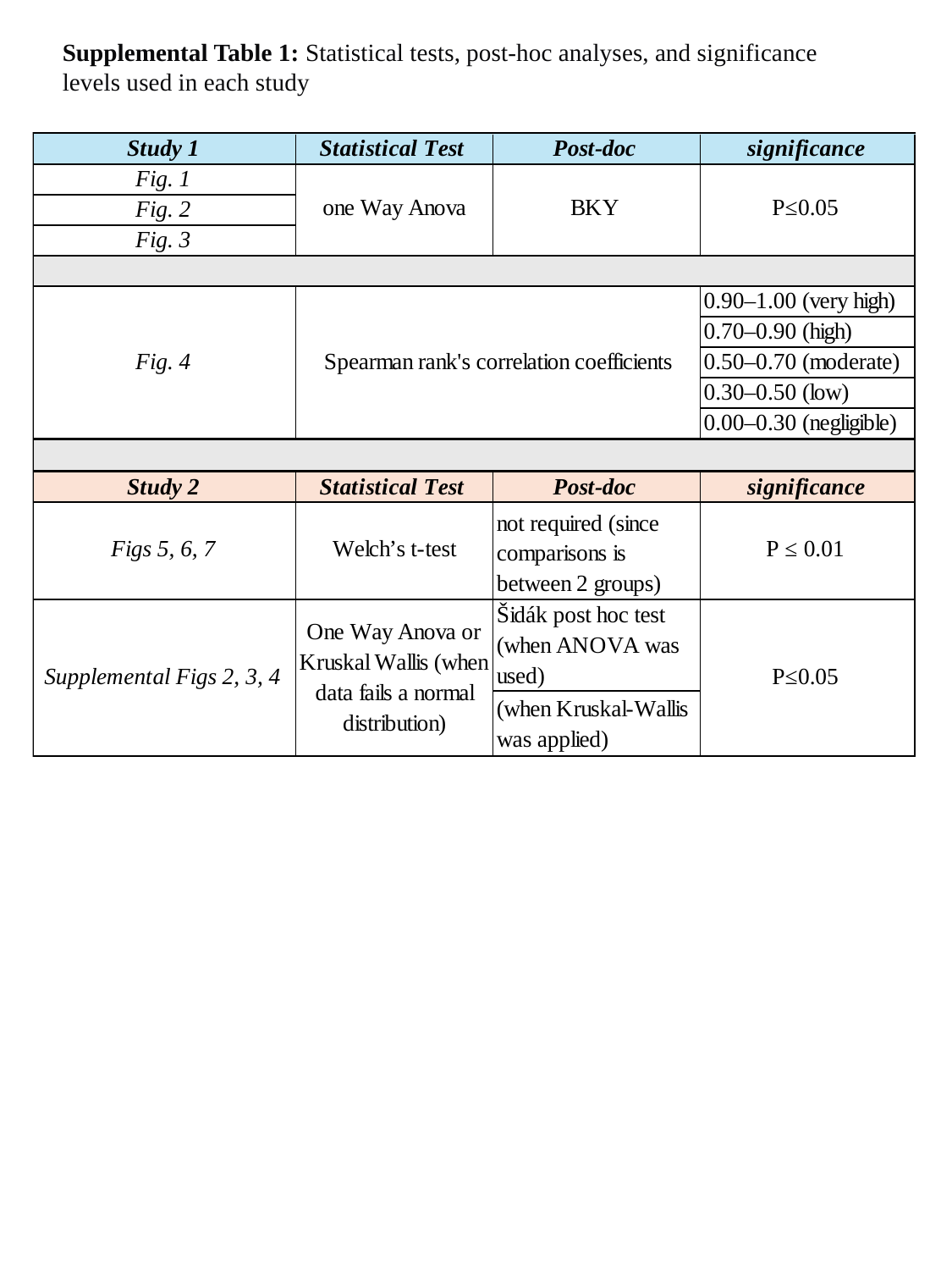

Supplemental Table 1: Statistical tests, post-hoc analyses, and significance levels used in each study
