# Supplementary material for: Dose-, duration- and age-dependent effects of zoledronic acid on bone structure and mechanical properties in growing rice rats
Source: Front Endocrinol (Lausanne). 2026 Jun 3;17:1772372. doi: 10.3389/fendo.2026.1772372 (PMC13271957; doi:10.3389/fendo.2026.1772372)
Supplement: Supplementary file 7 [file Presentation3.pptx]

## Slide 1
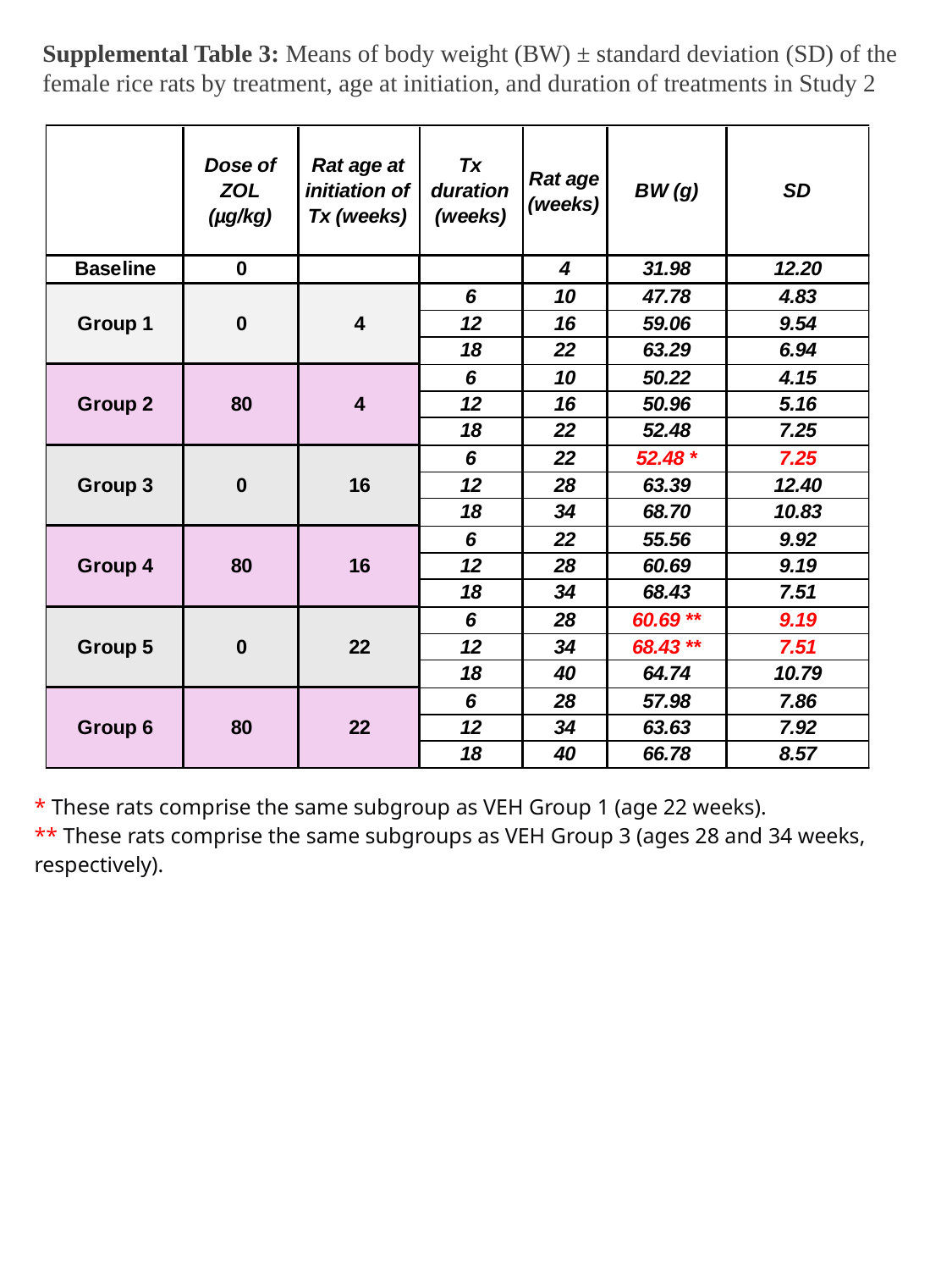

Supplemental Table 3: Means of body weight (BW) ± standard deviation (SD) of the female rice rats by treatment, age at initiation, and duration of treatments in Study 2
| \* These rats comprise the same subgroup as VEH Group 1 (age 22 weeks). \*\* These rats comprise the same subgroups as VEH Group 3 (ages 28 and 34 weeks, respectively). |
| --- |
