# Supplementary material for: Dose-, duration- and age-dependent effects of zoledronic acid on bone structure and mechanical properties in growing rice rats
Source: Front Endocrinol (Lausanne). 2026 Jun 3;17:1772372. doi: 10.3389/fendo.2026.1772372 (PMC13271957; doi:10.3389/fendo.2026.1772372)
Supplement: Supplementary file 14 [file DataSheet1.docx]

**SUPPLEMENTAL FIGURES**

**Supplemental Figure 1:** (**A**) *Study 1 Design*. 227 weanling female rice rats (4-week-old) were randomized to a baseline (BL) group (4 weeks; n =11, black diamond) or one of five treatment groups (n = 42–44/group). Rats received IV (tail vein) injections q4 weeks zoledronic acid (ZOL) 0 (saline), 8, 20, 50, or 125 µg/kg (dose groups color‑coded). Subgroups of 9-12 rats per dose were necropsied after 12, 18, 24, or 30 weeks of treatment (black diamonds). Treatment durations (weeks) are shown along the bottom of the schematic; corresponding rat ages are shown along the top. (**B**) *Study 2 Design.* 172 female rice rats were assigned to age- and treatment-defined groups. All ZOL treatments were administered at 80 µg/kg every 4 weeks (q4w). Groups: Baseline (BL) control (n = 12) rats necropsy at 4 weeks; Group 1 (n=32): VEH initiated at 4 wks, necropsies after 6, 12, 18 wks (n= 10-12/ subgroup); *Group 2* (n = 31): ZOL initiated at 4 weeks, necropsies after 6, 12, and 18 weeks of treatment (n= 10-11/subgroup); *Group 3* (VEH control for Group 4, n = 29): vehicle initiated at 16 weeks, necropsied at 6, 12, and 18 weeks (n = 7–12 per subgroup). *Group 4* (n = 39): ZOL initiated at 16 weeks, necropsies after 6, 12, and 18 weeks (ages 22, 29, 34 weeks; n = 12–15 per subgroup); *Group 5* (VEH control for Group 6, n = 25); vehicle initiated at 22 weeks, necropsied at age 28, 34 and 40 wks (7–8 per subgroups); *Group 6* (n = 31): ZOL initiated at 22 weeks, necropsies after 6, 12, and 18 weeks (ages 28, 34, 40 weeks; n = 7–12 per subgroup). Treatment durations (weeks) are shown at the bottom of the figure; corresponding rat ages are shown at the top. To further reduce animal use, the VEH subgroups corresponding to the 6- and 12-week VEH treatments in Group 5 were the same rats euthanized at 28 and 34 weeks of age in Group 3, respectively. To implement the 3Rs reduction principle, the 22-week VEH subgroup in Group 3 was matched to the 22-week VEH subgroup in Group 1. Further, the VEH subgroups corresponding to the 6- and 12-week VEH treatments in Group 5 were the same rats euthanized at 28 and 34 weeks of age in Group 3, respectively.

**Supplemental Figure 2**: ***pQCT measurements at the distal femur metaphysis in vehicle-treated rice rats across ages.*** Vehicle (VEH; saline) was given IV (tail vein) q4 weeks; rats (n = 10–15/group) were euthanized at 4, 10, 16, 22, 28, 34, and 40 weeks of age (4–10 wk: juvenile; 16–22 wk: young adult; 22–40 wk: mature adult). The ages shown on the x-axis represent the necropsy age of VEH-treated rats, calculated as the age at treatment initiation plus the duration of VEH administration. (**A**) total bone mineral content (BMC), (**B**) total volumetric BMD (vBMD), and (**C**) total metaphyseal area. Individual values are plotted as open circles; group means ± SD are indicated by red bars. Data were analyzed using one-way ANOVA with Holm–Šidák post hoc tests or, for non-normal distributions, the Kruskal–Wallis’s test with Dunn’s post hoc; *P*≤ 0.05 was considered significant. Superscript letters b–g indicate significant differences from age 10-, 16-, 22-, 28-, 34-, and 40-week rats, respectively.

**Supplemental Figure 3**: ***pQCT measurements at the femoral mid-diaphysis in vehicle-treated rice rats across ages.*** Vehicle (VEH; saline) was given IV (tail vein) q4 weeks; rats (n = 10–15/group) were euthanized at 4, 10, 16, 22, 28, 34, and 40 weeks of age (4–10 wk: juvenile; 16–22 wk: young adult; 22–40 wk: mature adult). The ages shown on the x-axis represent the necropsy age of VEH-treated rats, calculated as the age at treatment initiation plus the duration of VEH administration. (**A**) mid‑diaphyseal cortical bone mineral content (BMC), (**B**) cortical volumetric BMD (vBMD), (**C**) mid‑diaphyseal cortical area, (**D**) mid‑diaphyseal cortical thickness, (**E**) periosteal circumference, and (**F**) endocortical circumference. Individual values are plotted as open circles; group means ± SD are indicated by red bars. Data were analyzed using one-way ANOVA with Holm–Šidák post hoc tests or, for non-normal distributions, the Kruskal–Wallis test with Dunn’s post hoc; *P*≤ 0.05 was considered significant. Superscript letters b–g indicate significant differences from age 10-, 16-, 22-, 28-, 34-, and 40-week rats, respectively.

**Supplemental Figure 4:** ***Biomechanical properties at the femoral mid-diaphysis in rice rats in vehicle-treated rice rats across ages.*** Vehicle (VEH; saline) was given IV (tail vein) q4 weeks; rats (n = 10–15/group) were euthanized at 4, 10, 16, 22, 28, 34, and 40 weeks of age (4–10 wk: juvenile; 16–22 wk: young adult; 22–40 wk: mature adult). The ages shown on the x-axis represent the necropsy age of VEH-treated rats, calculated as the age at treatment initiation plus the duration of VEH administration. (**A**) stiffness (N/mm), (**B**) Ultimate load (N/mm), (**C**) work to fracture (mJ), (**D**) moment of inertia (mm^4^), (**E**) bending moment (N*mm), (**F**) elastic modulus (MPa), and (**G**) ultimate stress (MPa). Individual values are plotted as open circles; group means ± SD are indicated by red bars. Data were analyzed using one-way ANOVA with Holm–Šidák post hoc tests or, for non-normal distributions, the Kruskal–Wallis’s test with Dunn’s post hoc; P≤0.05 is considered statistically significant. Superscript letters b–g indicate significant differences from age 10-, 16-, 22-, 28-, 34-, and 40-week rats, respectively.
